# Supplementary material for: Identification of Novel Cyanopyridones and Pyrido[2,3-d]pyrimidines as Anticancer Agents with Dual VEGFR-2/HER-2 Inhibitory Action: Synthesis, Biological Evaluation and Molecular Docking Studies
Source: Pharmaceuticals (Basel). 2022 Oct 13;15(10):1262. doi: 10.3390/ph15101262 (PMC9655118; doi:10.3390/ph15101262)
Supplement: Supplementary file 1 [file pharmaceuticals-15-01262-s001.zip › Pharmaceuticals_1910885_SI.pdf]

Supporting Information

Identification of Novel Cyanopyridones and Pyrido[2,3-*d*]pyrimidines as anticancer agents with dual VEGFR-2/HER-2 inhibitory action: Synthesis, biological evaluation and molecular docking studies

Tarfah Al-Warhi, Al-Aliaa M. Sallam, Loah R Hemeda, Mahmoud A. El Hassab*, Nada Aljaeed, Ohoud J. Alotaibi, Ahmed S. Doghish, Mina Noshy, Wagdy M. Eldehna*, and Mona H. Ibrahim

Tables of Contents

1. **Physical and spectral data for target compounds** 2-6

2. **Cell viability assay** 7

3. **Statistical analysis** 7

4. **Molecular dynamics** 7-8

5. **NMR Spectra** 9-30

1. Physical and spectral data for target compounds

5.1.2.1. 6-Amino-2-oxo-4-phenyl-1-(3-(trifluoromethyl)phenyl)-1,2-dihydropyridine-3,5-dicarbonitrile (5a). **Yield** 90 %; **M.P.** 280-282 °C; **IR** (KBr, cm^{-1}): 3317, 3278 (NH_2), 2194 (C≡N), 1655 (C=O), 1631 (C=N), 1603 (C=C); **^{1}H NMR** (400 MHz, DMSO-d_6) δ (*ppm*): 7.51(d, 1H, H_4 CF_3-C_6H_4, J = 4.8 Hz), 7.53 (t, 1H, H_5 CF_3-C_6H_4, J = 2.8 Hz), 7.57 (d, 2H, $H_{2,6}$ C_6H_5, J = 4 Hz), 7.60 - 7.67 (t, 1H, H_4 C_6H_5, J = 8 Hz), 7.76 (d, 2H, $H_{3,5}$ C_6H_5, J = 8 Hz), 7.80 (s, 1H, H_2 CF_3-C_6H_4), 7.85 (d, 2H, H_6 CF_3-C_6H_4, J = 8 Hz), 8.26 (s, 2H, NH_2; exchangeable with D_2O); **^{13}C NMR** (100 MHz, DMSO-d_6) δ (*ppm*): 77.95, 84.48, 117.62, 118.01, 123.03, 125.74, 126.38, 126.38, 128.36, 129.05, 130.91, 131.45, 134.01, 135.94, 136.99, 158.24, 160.70, 161.19; **MS**, m/z: 380 (M^+, 100 %), 381 [$(M+1)^+$, 15.73 %], 382 [$(M+2)^+$, 1.46 %]; Anal. Calcd. For $C_{20}H_{11}F_3N_4O$ (381.1): C, 63.16; H, 2.92; N, 14.73, Found: C, 63.14; H, 2.91; N, 14.74.

5.1.2.2. 6-Amino-4-(4-hydroxyphenyl)-2-oxo-1-(3-(trifluoromethyl)phenyl)-1,2-dihydropyridine-3,5-dicarbonitrile (5b). **Yield** 88 %; **M.P.** 330 - 332 °C; **IR** (KBr, cm^{-1}): 3406 (OH), 3317, 3299 (NH_2), 2194 (2 C≡N), 1651 (C=O), 1631 (C=N), 1604 (C=C); **^{1}H NMR** (400 MHz, DMSO-d_6) δ (*ppm*): 4.45 (s, 2H, NH_2; exchangeable with D_2O), 6.95 (d, 2H, $H_{3,5}$ C_6H_4, J = 8 Hz), 7.40 (d, 2H, $H_{2,6}$ C_6H_4, J = 8 Hz), 7.73 (d, 1H, H_4 CF_3-C_6H_4, J = 8 Hz), 7.81 – 7.84 (t, 1H, H_5 CF_3-C_6H_4, J = 4 Hz), 7.93 – 8.00 (m, 2H, $H_{2,6}$ CF_3-C_6H_4), 10.13 (s, 1H, OH; exchangeable with D_2O); **^{13}C NMR** (100 MHz, DMSO-d_6) δ (*ppm*): 75.75, 88.06, 115.84, 116.59, 117.18, 122.94, 125.64, 126.74, 127.35, 130.35, 131.28, 131.96, 133.67, 135.36, 157.65, 159.97, 160.27, 161.93; **MS**, m/z: 396 (M^+, 100 %); Anal. Calcd. For $C_{20}H_{11}F_3N_4O_2$ (396.1): C, 60.61; H, 2.80; N, 14.14, Found: C, 60.63; H, 2.79; N, 14.13.

5.1.2.3. 6-Amino-4-(4-nitrophenyl)-2-oxo-1-(3-(trifluoromethyl)phenyl)-1,2-dihydropyridine-3,5-dicarbonitrile (5c). **Yield** 86 %; **M.P.** 229 - 230 °C; **IR** (KBr, cm^{-1}): 3375, 3321 (NH_2), 2206, 2191 (2 C≡N), 1680 (C=O), 1635 (C=N), 1612 (C=C) 1527, 1330 (NO_2); **^{1}H NMR** (400 MHz, DMSO-d_6) δ (*ppm*): 4.44 (s, 2H, NH_2; exchangeable with D_2O), 7.59 (d, 1H, H_4 CF_3-C_6H_4, J = 8 Hz), 7.70 (s, 1H, H_2 CF_3-C_6H_4), 7.75 – 7.76 (t, 1H, H_5 CF_3-C_6H_4, J = 4 Hz), 7.78 (d, 1H, H_6 CF_3-C_6H_4, J = 8 Hz), 7.80 (d, 2H, $H_{3,5}$ C_6H_4, J = 8. Hz), 8.40 (d, 2H, $H_{2,6}$ C_6H_4, J = 8 Hz); **^{13}C NMR** (100 MHz, DMSO-d_6) δ

(*ppm*): 79.18, 81.62, 118.07, 118.40, 123.05, 124.27, 125.76, 126.86, 126.90, 130.12, 130.94, 131.21, 134.19, 137.81, 148.58, 157.88, 158.26, 161.61; **MS**, m/z: 425 (M^+, 16.37 %); Anal. Calcd. For $C_{20}H_{10}F_3N_5O_3$ (425.1): C, 56.48; H, 2.37; N, 16.47, Found: C, 56.50; H, 2.38; N, 16.46.

5.1.2.4. 6-Amino-4-(4-chlorophenyl)-2-oxo-1-(3-(trifluoromethyl)phenyl)-1,2-dihydropyridine-3,5-dicarbonitrile (5d). **Yield** 95 %; **M.P.** 324 - 326 °C; **IR** (KBr, cm^{-1}): 3303, 3286 (NH_2), 2210, 2198 (2C≡N), 1689 (C=O), 1631 (C=N), 1606 (C=C); **^{1}H NMR** (400 MHz, DMSO-d_6) δ (*ppm*): 7.57 (d, 2H, $H_{2,6}$ C_6H_4, J = 8 Hz), 7.69 (t, 1H, H_5 CF_3-C_6H_4, J = 2.4 Hz), 7.70 (d, 2H, $H_{3,5}$ C_6H_4, J = 8 Hz), 7.75 (s, 1H, H_2 CF_3-C_6H_4), 7.82 (d, 1H, H_4 CF_3-C_6H_4, J = 8 Hz), 7.92 (d, 1H, H_6 CF_3-C_6H_4, J = 8 Hz), 8.13 (s, 2H, NH_2; exchangeable with D_2O); **^{13}C NMR** (100 MHz, DMSO-d_6) δ (*ppm*): 94.40, 102.54, 120.70, 123.05, 125.89, 126.57, 127.74, 130.39, 133.07, 133.71, 137.89, 138.73, 147.56, 150.78, 150.95, 156.37, 158,88, 161.41; **MS**, m/z: 414 (M^+, 100 %), 415 [$(M+1)^+$, 0.21 %]; Anal. Calcd. For $C_{20}H_{10}ClF_3N_4O$ (414): C, 57.92; H, 2.43; N, 13.51, Found: C, 57.93; H, 2.42; N, 13.50.

5.1.2.5. 6-Amino-4-(2,4-dichlorophenyl)-2-oxo-1-(3-(trifluoromethyl)phenyl)-1,2-dihydropyridine-3,5-dicarbonitrile (5e). **Yield** 80 %; **M.P.** 239 - 241°C; **IR** (KBr, cm^{-1}): 3325, 3282 (NH_2), 2194 (2C≡N), 1643 (C=O), 1632 (C=N), 1603 (C=C); **^{1}H NMR** (400 MHz, DMSO-d_6) δ (*ppm*): 6.13 (s, 2H, NH_2; exchangeable with D_2O), 7.50 (d, 1H, H_6 C_6H_3, J = 8 Hz), 7.57 (d, 1H, H_5 C_6H_3, J = 4 Hz), 7.61 (s, 1H, H_3 C_6H_3), 7.63 (d, H, H_4 CF_3-C_6H_4, J = 10 Hz), 7.72 – 7.76 (t, H, H_5 CF_3-C_6H_4, J = 8 Hz), 7.78 (d, 1H, H_6 CF_3-C_6H_4, J = 8 Hz), 7.87 (s, 1H, H_2 CF_3-C_6H_4); **^{13}C NMR** (100 MHz, DMSO-d_6) δ (*ppm*): 80.08, 80.76, 118.14, 123.13, 125.37, 125.85, 126.98, 128.46, 129.78, 130.46, 131.01, 131.60, 132.64, 134.37, 134.96, 135.25, 138.29, 156.19, 158.33, 161.94; **MS**, m/z: 448 (M^+, 30.62); Anal. Calcd. For $C_{20}H_9Cl_2F_3N_4O$ (448): C, 53.48; H, 2.02; N, 12.47, Found: C, 53.50; H, 2.01; N, 12.46.

5.1.2.6. 6-Amino-2-oxo-1-(3-(trifluoromethyl)phenyl)-4-(3,4,5-trimethoxyphenyl)-1,2-dihydropyridine-3,5-dicarbonitrile (5f). **Yield** 75 %; **M.P.** 195 – 197 °C; **IR** (KBr, cm^{-1}): 3387, 3329 (NH_2), 2210, 2191 (2C≡N), 1674 (C=O), 1643 (C=N), 1624 (C=C); **^{1}H NMR** (400 MHz, DMSO-d_6) δ (*ppm*): 3.77 (s, 3H, 4-CH_3O-Hs), 3.85 (s, 6H, 3,5-di (CH_3O) -

Hs), 6.84 (s, 2H, $H_{2,6}$ C_6H_2), 7.61 (d, 1H, H_4 CF_3-C_6H_4, *J* = 6 Hz), 7.74 (s, 1H, H_2 CF_3-C_6H_4), 7.76 – 7.78 (t, 1H, H_5 CF_3-C_6H_4, *J* = 4 Hz), 7.84 (d, 1H, H_6 CF_3-C_6H_4, *J* = 8 Hz); **^{13}C NMR** (100 MHz, DMSO-*d*$_6$) δ (*ppm*): 56.74, 60.66, 78.12, 80.01, 106.17, 118.00, 118.34, 123.28, 125.73, 126.68, 130.79, 131.11, 131.68, 134.01, 137.09, 138.89, 153.25, 158.34, 160.27, 161.29; **MS**, m/z: 470 (M^+, 43.10); Anal. Calcd. For $C_{23}H_{17}F_3N_4O_4$ (470.1): C, 58.73; H, 3.64; N, 11.91, Found: C, 58.75; H, 3.63; N, 11.90.

5.1.3.1. 5-(4-Hydroxyphenyl)-4,7-dioxo-8-(3-(trifluoromethyl)phenyl)-3,4,7,8-tetrahydropyrido[2,3-d]pyrimidine-6-carbonitrile (6a). **Yield** 68 %; **M.P.** 300 – 302 °C; **IR** (KBr, cm^{-1}): 3316 (NH), 2215 (C≡N), 1676, 1656 (2C=O), 1634 (C=N), 1603 (C=C); **^{1}H NMR** (400 MHz, DMSO-*d*$_6$) δ (*ppm*): 6.95 (d, 2H, $H_{3,5}$ C_6H_4, *J* = 8 Hz), 7.40 (d, 2H, $H_{2,6}$ C_6H_4, *J* = 8 Hz), 7.73 (d, 1H, H_4 CF_3-C_6H_4, *J* = 8 Hz), 7.81 – 7.84 (t, 1H, H_5 CF_3-C_6H_4, *J* = 4 Hz), 7.91 (m, 2H, $H_{2,6}$ CF_3-C_6H_4), 7.99 (s, 1H, H_2 pyrimidine), 8.32 (s, 1H, NH; exchangeable with D_2O), 10.13 (s, 1H, OH; exchangeable with D_2O); **^{13}C NMR** (100 MHz, DMSO-*d*$_6$) δ (*ppm*): 75.77, 88.09, 115.85, 116.58, 122.93, 125.33, 125.64, 126.74, 127.38, 130.35, 131.29, 131.96, 133.66, 135.36, 157.66, 159.97, 160.27, 161.94, 163.54; **MS**, m/z: 424 (M^+, 38.61%), 425 [$(M+1)^+$, 21.83 %], 426 [$(M+2)^+$, 16.89 %]; Anal. Calcd. For $C_{21}H_{11}F_3N_4O_3$ (424.1): C, 59.44; H, 2.61; N, 13.20; Found: C, 59.42; H, 2.60; N, 13.21.

5.1.3.2. 5-(4-Chlorophenyl)-4,7-dioxo-8-(3-(trifluoromethyl)phenyl)-3,4,7,8-tetrahydropyrido[2,3-d]pyrimidine-6-carbonitrile (6b). **Yield** 75 %; **M.P.** 350 – 352 °C; **IR** (KBr, cm^{-1}): 3330 (NH), 2217 (C≡N), 1689, 1670 (2C=O), 1623 (C=N), 1603 (C=C); **^{1}H NMR** (400 MHz, DMSO-*d*$_6$) δ (*ppm*): 7.41 (d, 2H, $H_{3,5}$ C_6H_4 , *J* = 8 Hz), 7.45 (d, 1H, H_4 CF_3-C_6H_4, *J* = 8 Hz), 7.59 – 7.68 (t, 1H, H_5 CF_3-C_6H_4, *J* = 4 Hz), 7.80 (d, 2H, $H_{2,6}$ C_6H_4 , *J* = 8 Hz), 7.83 (d, 1H, H_6 CF_3-C_6H_4, *J* = 8 Hz), 8.17 (s, H, H_2 CF_3-C_6H_4), 8.33 (s, H, H_2 pyrimidine), 9.45 (s, 1H, NH; exchangeable with D_2O); **^{13}C NMR** (100 MHz, DMSO-*d*$_6$) δ (*ppm*): 75.91, 88.42, 116.08, 116.65, 120.21, 122.92, 125.62, 126.67, 127.45, 129.45, 130.36, 131.02, 131.98, 132.05, 133.95, 135.73, 157.66, 160.00, 160.78; **MS**, m/z: 442 (M^+, 19.98%); Anal. Calcd. For $C_{21}H_{10}ClF_3N_4O_2$ (442.1): C, 56.97; H, 2.28; N, 12.65; Found: C, 56.99; H, 2.27; N, 12.66.

5.1.4.1. 2-Methyl-4,7-dioxo-5-phenyl-8-(3-(trifluoromethyl)phenyl)-3,4,7,8-tetrahydropyrido[2,3-d]pyrimidine-6-carbonitrile (7a). **Yield** 73 %; **M.P.** 308 - 310 °C; **IR** (KBr, cm^{-1}): 3299 (NH), 2214 (C≡N), 1689, 1668 (2C=O), 1623 (C=N), 1610 (C=C); **^{1}H NMR** (400 MHz, DMSO-*d*$_6$) *δ* (*ppm*): 1.93 (s, 3H, CH_3-Hs (Pyrimidine)), 7.56 – 7.61 (m, 5 H, $H_{2,3,4,5,6}$ C_6H_5), 7.75 (d, 1H, H_4 CF_3-C_6H_4, *J* = 8 Hz), 7.82 (t, 1H, H_5 CF_3-C_6H_4, *J* = 4 Hz), 7.86 (s, 1H, H_2 CF_3-C_6H_4), 7.92 (d, 1H, H_6 CF_3-C_6H_4, *J* = 10 Hz), 8.09 (s, 1H, NH; exchangeable with D_2O); **^{13}C NMR** (100 MHz, DMSO-*d*$_6$) *δ* (*ppm*): 21.52, 75.98, 88.44, 116.19, 116.79, 126.69, 127.43, 127.46, 128.34, 129.24, 130.88, 131.34, 131.66, 132.04, 133.60, 135.12, 135.26, 157.66, 160.13, 162.00; **MS**, m/z: 422 (M^+, 7.91 %); Anal. Calcd. For $C_{22}H_{13}F_3N_4O_2$ (422.1): C, 62.56; H, 3.10; N, 13.27, Found: C, 62.58; H, 3.09; N, 13.28.

5.1.4.2. 5-(4-Chlorophenyl)-2-methyl-4,7-dioxo-8-(3-(trifluoromethyl)phenyl)-3,4,7,8-tetrahydropyrido[2,3-d]pyrimidine-6-carbonitrile (7b). **Yield** 62 %; **M.P.** 330 - 332 °C; **IR** (KBr, cm^{-1}): 3224 (NH), 2222 (C≡N), 1682, 1663 (2C=O), 1632 (C=N), 1604 (C=C); **^{1}H NMR** (400 MHz, DMSO-*d*$_6$) *δ* (*ppm*): 2.19 (s, 3H, CH_3-Hs (Pyrimidine)), 7.58 (d, 2 H, $H_{2,6}$ C_6H_4, *J* = 6 Hz), 7.70 (d, 2H, $H_{3,5}$ C_6H_4, *J* = 8 Hz), 7.75 (s, 1H, H_2 CF_3-C_6H_4), 7.82 – 7.86 (t, 1H, H_5 CF_3-C_6H_4, *J* = 6 Hz), 7.89 (d, H, H_4 CF_3-C_6H_4, *J* = 8 Hz), 7.95 (d, 2H, H_6 CF_3-C_6H_4, *J* = 8 Hz), 8.14 (s, 1H, NH; exchangeable with D_2O); **^{13}C NMR** (100 MHz, DMSO-*d*$_6$) *δ* (*ppm*): 22.38, 76.02, 88.38, 116.02, 116.65, 122.92, 125.63, 126.67, 127.27, 129.38, 130.27, 131.78, 133.89, 134.91, 135.89, 137.40, 157.66, 159.77, 160.89, 163.78; **MS**, m/z: 456 (M^+, 34.11 %); Anal. Calcd. For $C_{22}H_{12}ClF_3N_4O_2$ (456.1): C, 57.85; H, 2.65; N, 12.27, Found: C, 57.87; H, 2.65; N, 12.26.

5.1.4.3. 5-(2,4-Dichlorophenyl)-2-methyl-4,7-dioxo-8-(3-(trifluoromethyl)phenyl)-3,4,7,8-tetrahydropyrido[2,3-d]pyrimidine-6-carbonitrile (7c). **Yield** 90 %; **M.P.** 333 - 335 °C; **IR** (KBr, cm^{-1}): 3202 (NH), 2226 (C≡N), 1682 (2C=O), 1625 (C=N), 1601 (C=C); **^{1}H NMR** (400 MHz, DMSO-*d*$_6$) *δ* (*ppm*): 2.20 (s, 3H, CH_3-Hs (Pyrimidine)), 7.50 (d, H, H_4 CF_3-C_6H_4), 7.57 (d, H, H_6 CF_3-C_6H_4), 7.70- 7.90 (m, 3H, $H_{5,6}$ C_6H_3, H_5 CF_3-C_6H_4), 7.93 (s, 1H, H_3 C_6H_3), 7.94 (s, 1H, H_2 CF_3-C_6H_4), 12.88 (s, 1H, NH; exchangeable with D_2O); **^{13}C NMR** (100 MHz, DMSO-*d*$_6$) *δ* (*ppm*): 22.44, 76.49, 89.13, 115.32, 115.98, 126.52, 127.56, 128.17, 128.82, 130.03, 130.76, 131.51, 132.39, 134.73,

135.09, 156.48, 158.79, 159.75, 164.11, 164.23; **MS**, m/z: 490 (M^+, 14.97 %); Anal. Calcd. For $C_{22}H_{11}Cl_2F_3N_4O_2$ (490): C, 53.79; H, 2.26; N,11.41, Found: C, 53.80; H, 2.27; N, 11.40.

5.1.4.4. 4-(6-Cyano-2-methyl-4,7-dioxo-8-(3-(trifluoromethyl)phenyl)-3,4,7,8-tetrahydropyrido[2,3-d]pyrimidin-5-yl)phenyl acetate (7d). **Yield** 67 %; **M.P.** 320 - 322 °C; **IR** (KBr, cm^{-1}): 3206 (NH), 2225 (C≡N), 1773, 1686 (3C=O), 1622 (C=N), 1593 (C=C); **^{1}H NMR** (400 MHz, DMSO-d_6) δ (*ppm*): 2.18 (s, 3H, CH_3CO), 2.34 (s, 3H, CH_3-Hs (Pyrimidine)), 7.27 (d, 2H, $H_{2,6}$ C_6H_4, J = 8 Hz), 7.39 (d, 1H, $H_{3,5}$ C_6H_4, J = 8 Hz), 7.42 (d, 1H, H_4 CF_3-C_6H_4, J = 7 Hz), 7.69 (d, 1H, H_6 CF_3-C_6H_4, J = 8 Hz), 7.79 – 7.94 (m, 1H, $H_{2,5}$ CF_3-C_6H_4), 12.77 (s, 1H, NH; exchangeable with D_2O); **^{13}C NMR** (100 MHz, DMSO-d_6) δ (*ppm*): 21.45, 22.34, 101.45, 103.74, 115.56, 121.82, 125.93, 125.97, 126.26, 128.97, 130.06, 130.75, 133.56, 133.85, 137.57, 151.29, 157.56, 158.88, 159.87, 160.04, 163.71, 169.45 ; **MS**, m/z: 480 (M^+, 27.20 %); Anal. Calcd. For $C_{24}H_{15}F_3N_4O_4$ (480): C, 60.00; H, 3.15; N, 11.66; Found: C, 59.98; H, 3.14; N, 11.65.

5.1.4.5. 2-Methyl-4,7-dioxo-8-(3-(trifluoromethyl)phenyl)-5-(3,4,5-trimethoxyphenyl)-3,4,7,8-tetrahydropyrido[2,3-d]pyrimidine-6-carbonitrile (7e). **Yield** 89 %; **M.P.** 150 - 152 °C; **IR** (KBr, cm^{-1}): 3209 (NH), 2224 (C≡N), 1674 (2C=O), 1630 (C=N), 1589 (C=C); **^{1}H NMR** (400 MHz, DMSO-d_6) δ (*ppm*): 2.19 (s, 3H, CH_3-Hs (Pyrimidine)), 3.77 (s, 3H, 4-CH_3O-H), 3.86 (s, 6H, 3,5-di (CH_3O) - Hs), 6.87 (s, 2H, $H_{2,6}$ C_6H_2), 7.66 (d, 1H, H_4 CF_3-C_6H_4, J = 8 Hz), 7.80 (s, 1H, H_2 CF_3-C_6H_4), 7.81 (d, 1H, H_6 CF_3-C_6H_4, J = 7 Hz), 7.88 – 7.90 (t, 1H, H_5 CF_3-C_6H_4, J = 6 Hz); 12.66 (s, 1H, NH; exchangeable with D_2O); **^{13}C NMR** (100 MHz, DMSO-d_6) δ (*ppm*):22.32, 56.49, 56.62, 60.51, 75.85, 88.28, 101.52, 103.74, 106.06, 115.49, 130.94, 131.92, 133.41, 137.51, 138.18. 139.30, 152.81, 157.32, 158.41, 159.92, 161.41, 162.01, 163.61; **MS**, m/z: 512 (M^+, 34.33 %); Anal. Calcd. For $C_{25}H_{19}F_3N_4O_5$ (512.1): C, 58.60; H, 3.74; N, 10.93; Found: C, 58.62; H, 3.74; N, 10.94.

2. Cell viability assay

The MTT assay was used to determine the effects of the newly synthesized compounds on cell viability [49-51]. In a nutshell, 1×10^4 cells/well were seeded in a 96-well plate and allowed to grow for 24 hours. After 24 hours, the original medium was discarded, and a new compounds-containing medium was added. After 48 hours, the media was changed out and 100 µL of MTT (10 µL from (MTT) stock solution (5 mg/ml in PBS) in 100 µL media) was added to each well, and the plates were incubated at 37° C for 4 hours. After adding 100 µL of acidified isopropanol to each well, the formed formazan crystals were dissolved, and the absorbance was read at 570 nm using an ELISA microplate reader (Epoc-2C micro-plate reader, Bio Tek, VT, USA). There were three separate runs of every experiment. Half-maximal inhibitory concentrations (IC_{50}) were determined by fitting sigmoidal dose-response curves.

3. Statistical Analysis

GraphPad Prism 8.0 (San Diego, CA, USA) analyzed all results. All results (three independent experiments) were shown as means ± standard deviation. The significance of the differences in the outcomes of all groups was examined using ANOVA and Tukey's multiple comparisons tests. Statistical significance was defined as a $p < 0.05$

4. Molecular dynamics

In this work, six molecular dynamic simulations (MDS) were conducted for 150000ps using GROMACS 5.1.1 software. The two free enzymes and the retrieved docking coordinates of the same enzymes bound compound 5e, TAK-285 and Sorafenib were used as input structures for the molecular dynamics. The receptor and ligand topologies were generated by PDB2gmx (embedded in GROMACS) and GlycoBioChem PRODRG2 Server respectively, both under GROMOS96 force field was implemented to generate the ligand topologies using the. After rejoining ligands and receptor topologies to generate six systems, the typical molecular dynamics scheme of GROMACS was applied for all the systems. This include, solvation, neutralization, energy minimization under GROMOS96 43a1 force field and two stages of equilibration (NVT and NPT).

Finally, unrestricted production stage of 150ns was applied for the six systems with particle mesh ewald (PME) method implemented to compute the long-range electrostatic values using 12 Å cut-off and 12 Å Fourier spacing. The stability of the complexes was judged using RMSD and RMSF values calculated from the MDS trajectories from the production step.

MMPBSA calculations

The following equation was implemented to calculate the binding free energies:

$$\Delta G_{(Binding)} = G_{(Complex)} - G_{(Receptor)} - G_{(Ligand)}$$

Where $G_{(Complex)}$ is the total free energy of the protein−ligand complex and $G_{(Receptor)}$ and $G_{(Ligand)}$ are the total free energies of the isolated protein and ligand in solvent, respectively. The total free energy of any of the three mentioned entities (complex, receptor and ligand) were calculated for all MD trajectories from its molecular mechanics potential energy plus the energy of the solvation, using the g_mmpbsa package implemented in the GROMACS software. Individual energies along with the values of standard deviations were calculated and then summed together to yield the average total free energy of each component. Finally, to calculate the binding-free energy, the total free energy of the receptor and the ligand were subtracted from the total free energy of the complex.

Mona Hussein-A10c-AS-proton
Mona Hussein-A10c-AS-proton

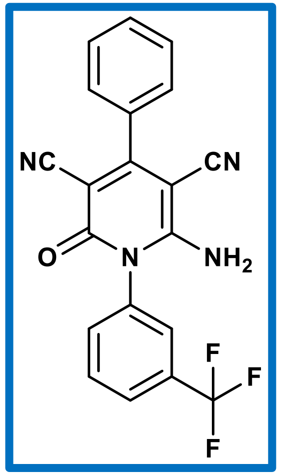

5a

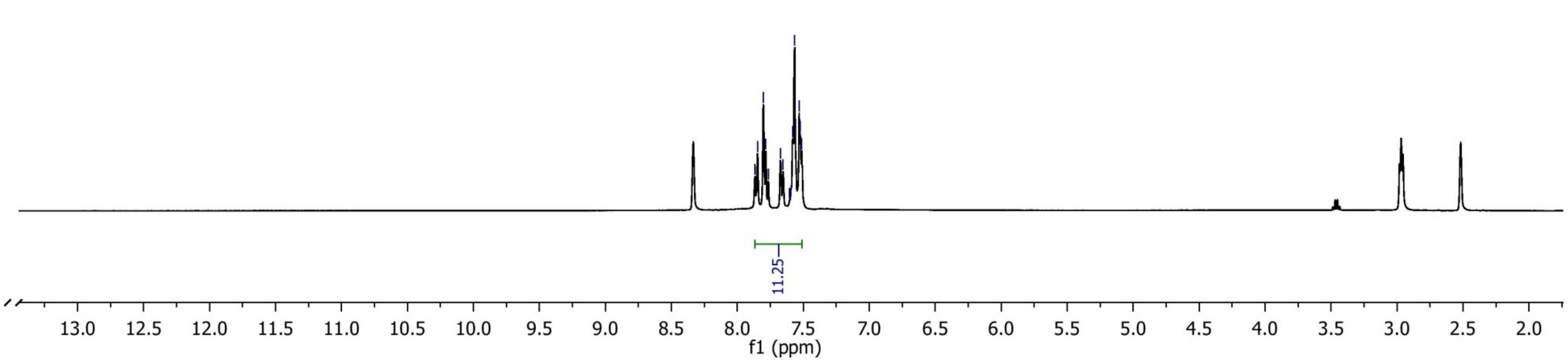

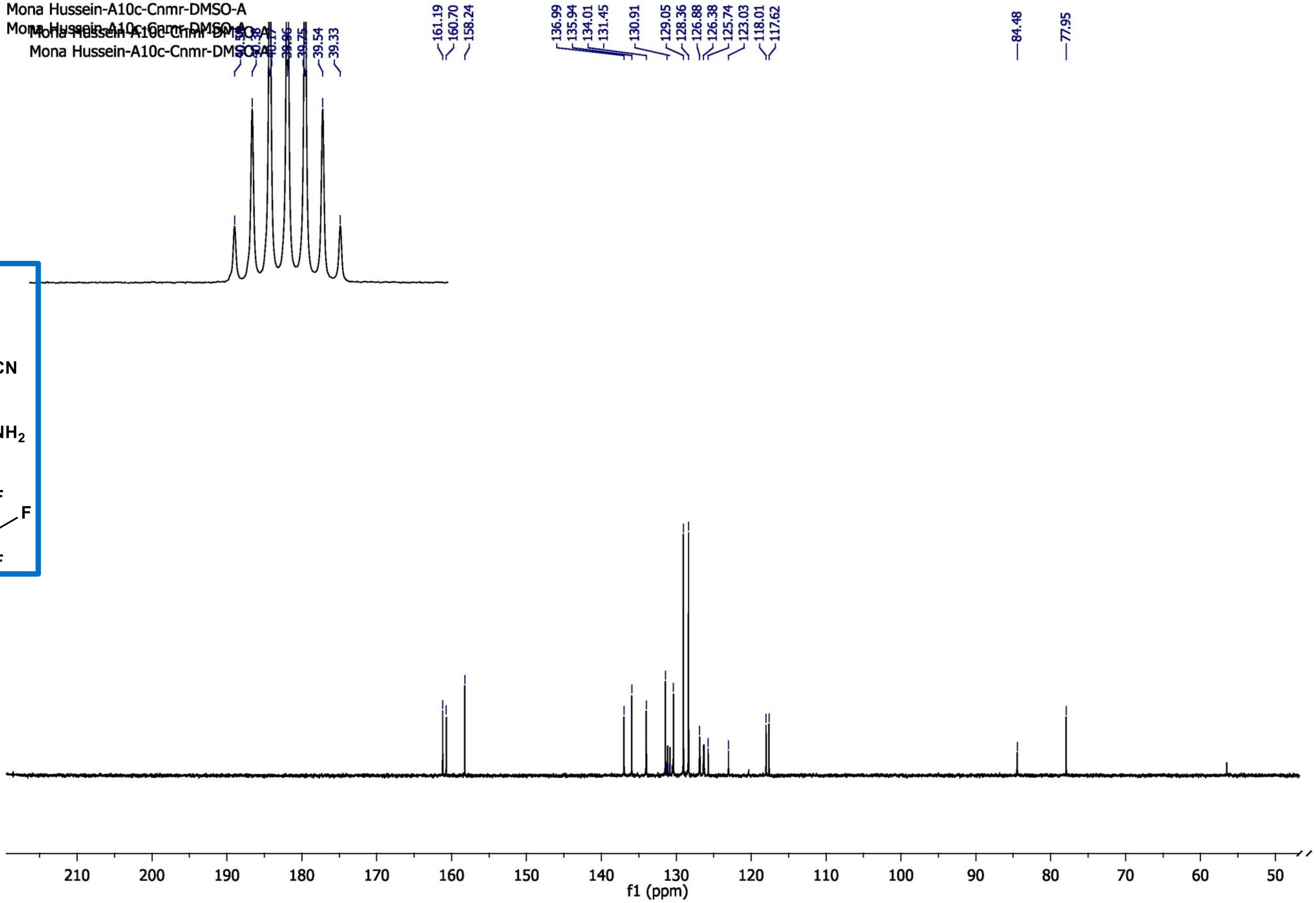
Mona Hussein-A10c-Cnmr-DMSO-A
39.96
39.75
39.54
39.33
161.19
160.70
158.24
136.99
135.94
134.01
131.45
130.91
129.05
128.36
126.88
126.38
125.74
123.03
118.01
117.62
84.48
77.95
210 200 190 180 170 160 150 140 130 120 110 100 90 80 70 60 50
f1 (ppm)

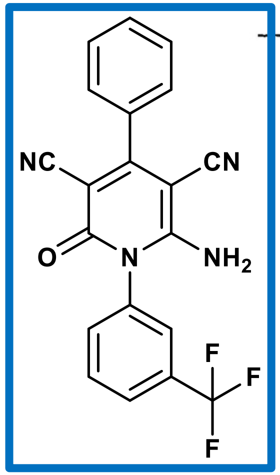
NC
CN
O
N
NH2
F
F
F

5a

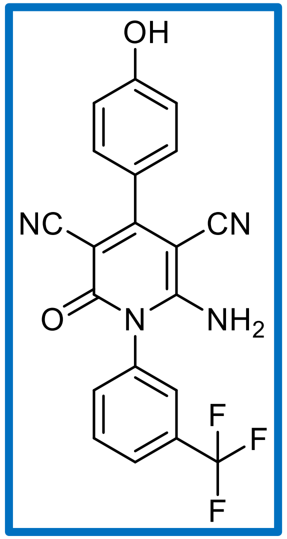
OH
NC
CN
O
N
NH2
F
F
F

5b

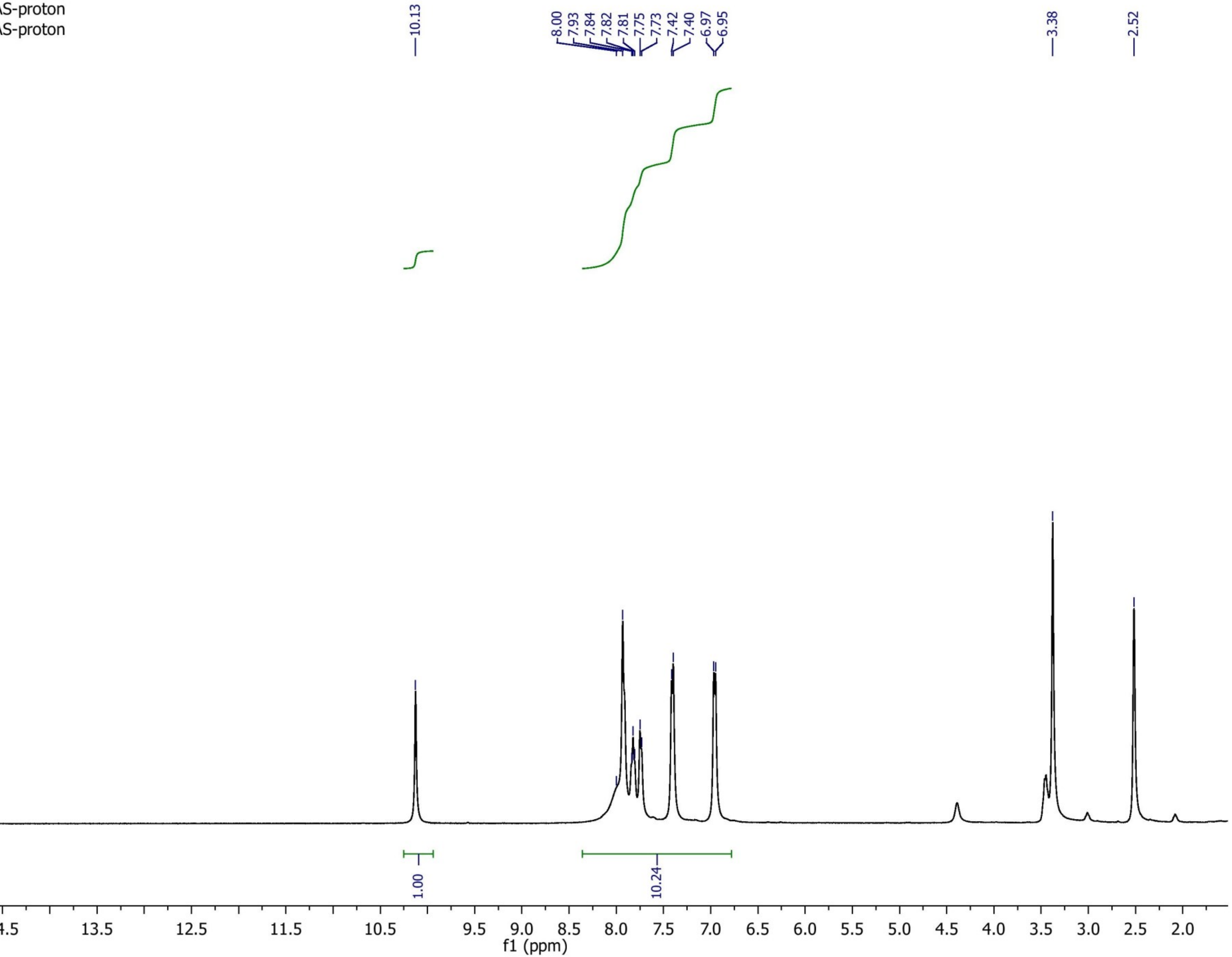
Mona Hussein-A11c-AS-proton
Mona Hussein-A11c-AS-proton
10.13
8.00
7.93
7.84
7.82
7.81
7.75
7.73
7.42
7.40
6.97
6.95
3.38
2.52
1.00
10.24
15.5
14.5
13.5
12.5
11.5
10.5
9.5
9.0
8.5
8.0
7.5
7.0
6.5
6.0
5.5
5.0
4.5
4.0
3.5
3.0
2.5
2.0
f1 (ppm)

Mona Hussin-A11C-D2O-AF
Mona Hussin-A11C-D2O-AF

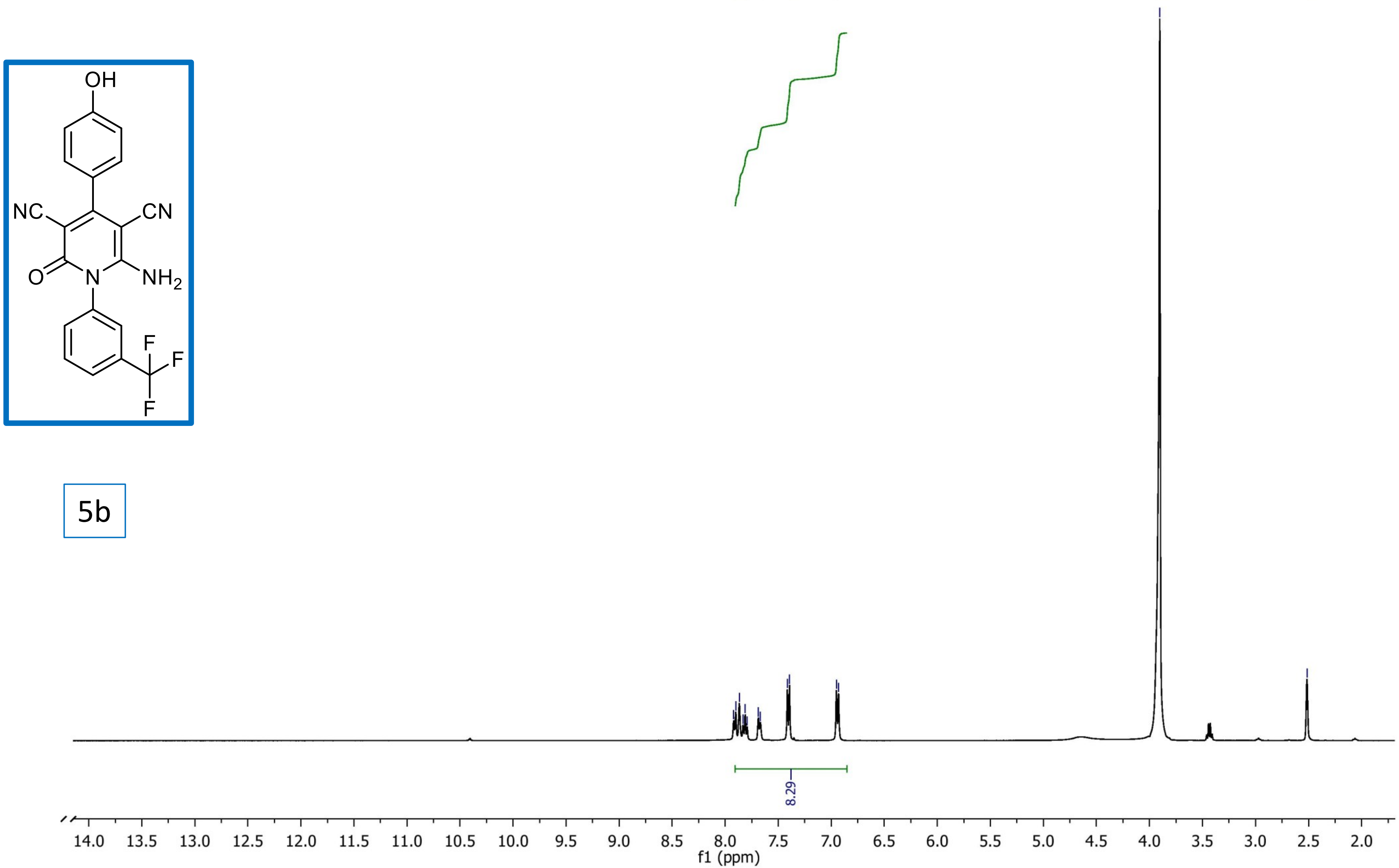

5b

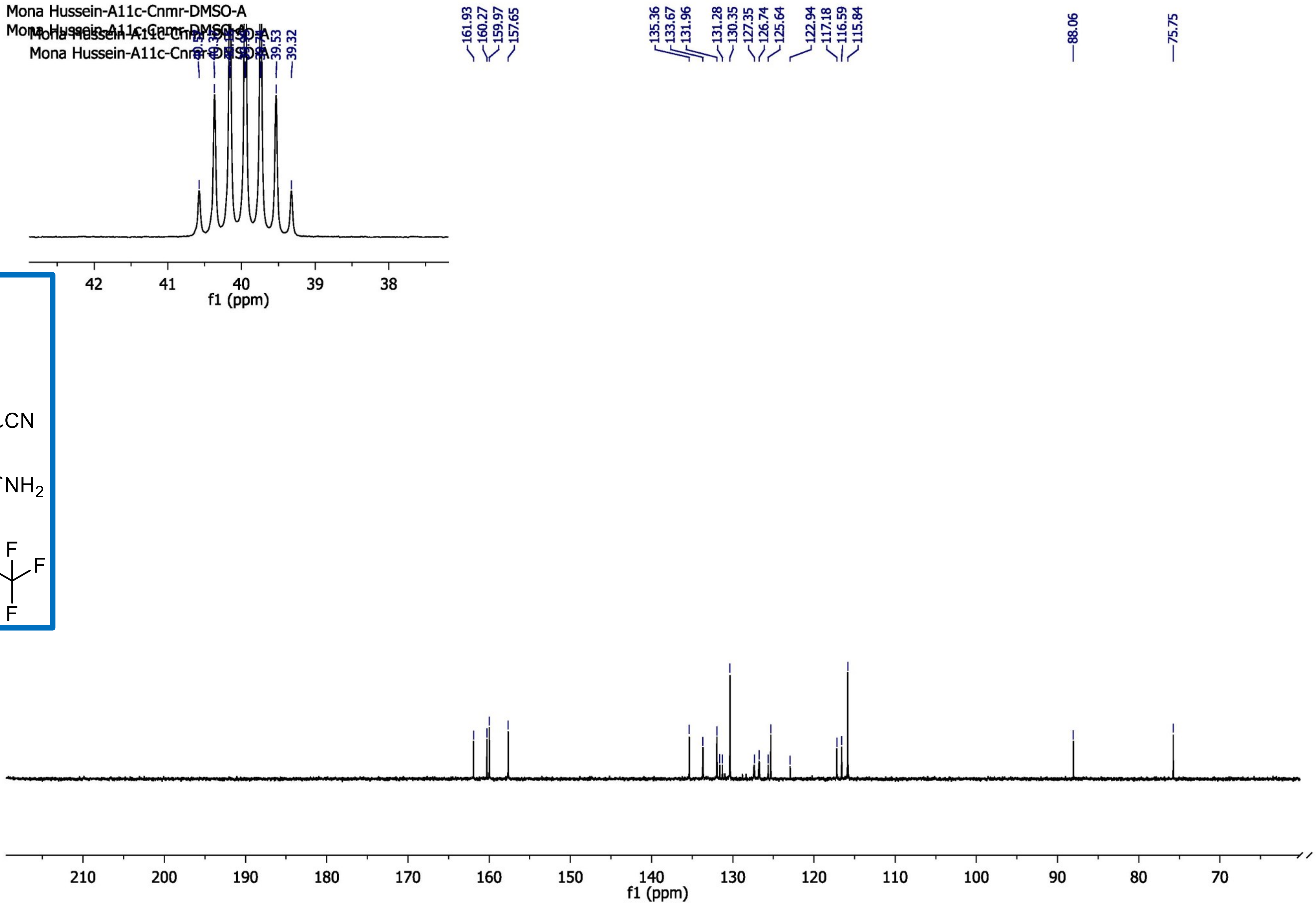
Mona Hussein-A11c-Cnmr-DMSO-A
Mona Hussein-A11c-Cnmr-DMSO-A
39.53
39.32
42
41
40
39
38
f1 (ppm)
161.93
160.27
159.97
157.65
135.36
133.67
131.96
131.28
130.35
127.35
126.74
125.64
122.94
117.18
116.59
115.84
88.06
75.75
210
200
190
180
170
160
150
140
130
120
110
100
90
80
70
f1 (ppm)

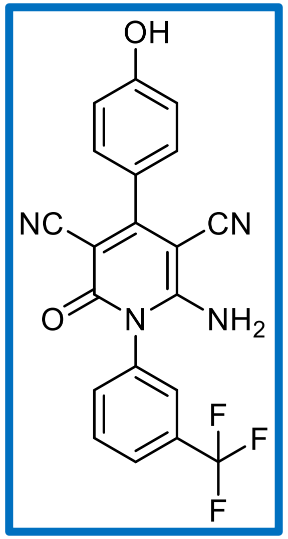
OH
NC
CN
O
N
NH_2
F
F
F

5b

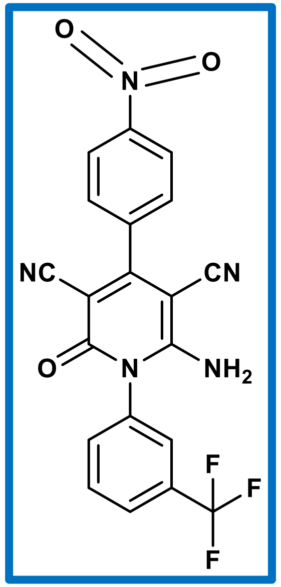
O
N
O
NC
CN
O
N
NH2
F
F
F

5c

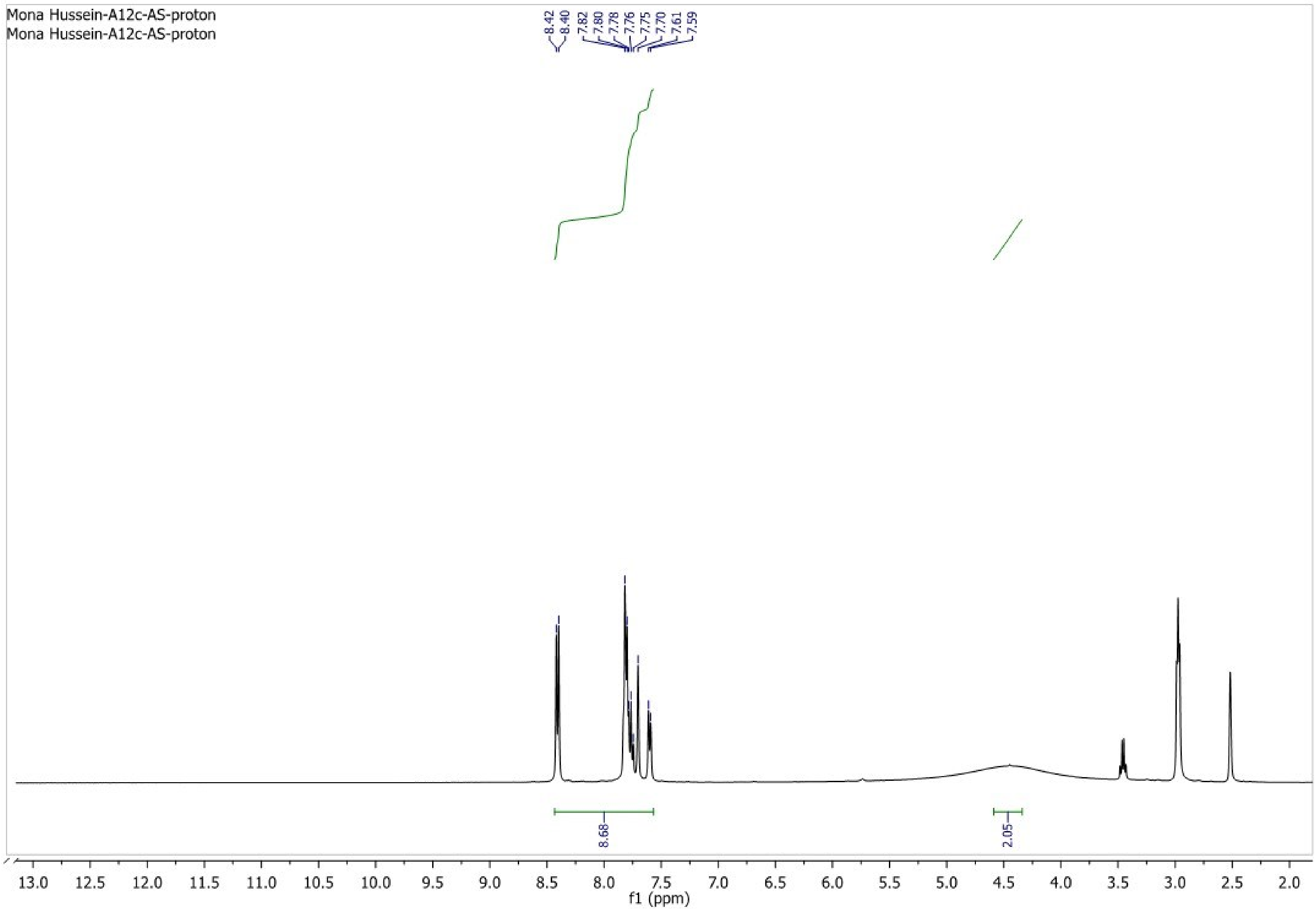
Mona Hussein-A12c-AS-proton
Mona Hussein-A12c-AS-proton
8.42
8.40
7.82
7.80
7.78
7.76
7.75
7.70
7.61
7.59
8.68
2.05
13.0
12.5
12.0
11.5
11.0
10.5
10.0
9.5
9.0
8.5
8.0
7.5
7.0
6.5
6.0
5.5
5.0
4.5
4.0
3.5
3.0
2.5
2.0
f1 (ppm)

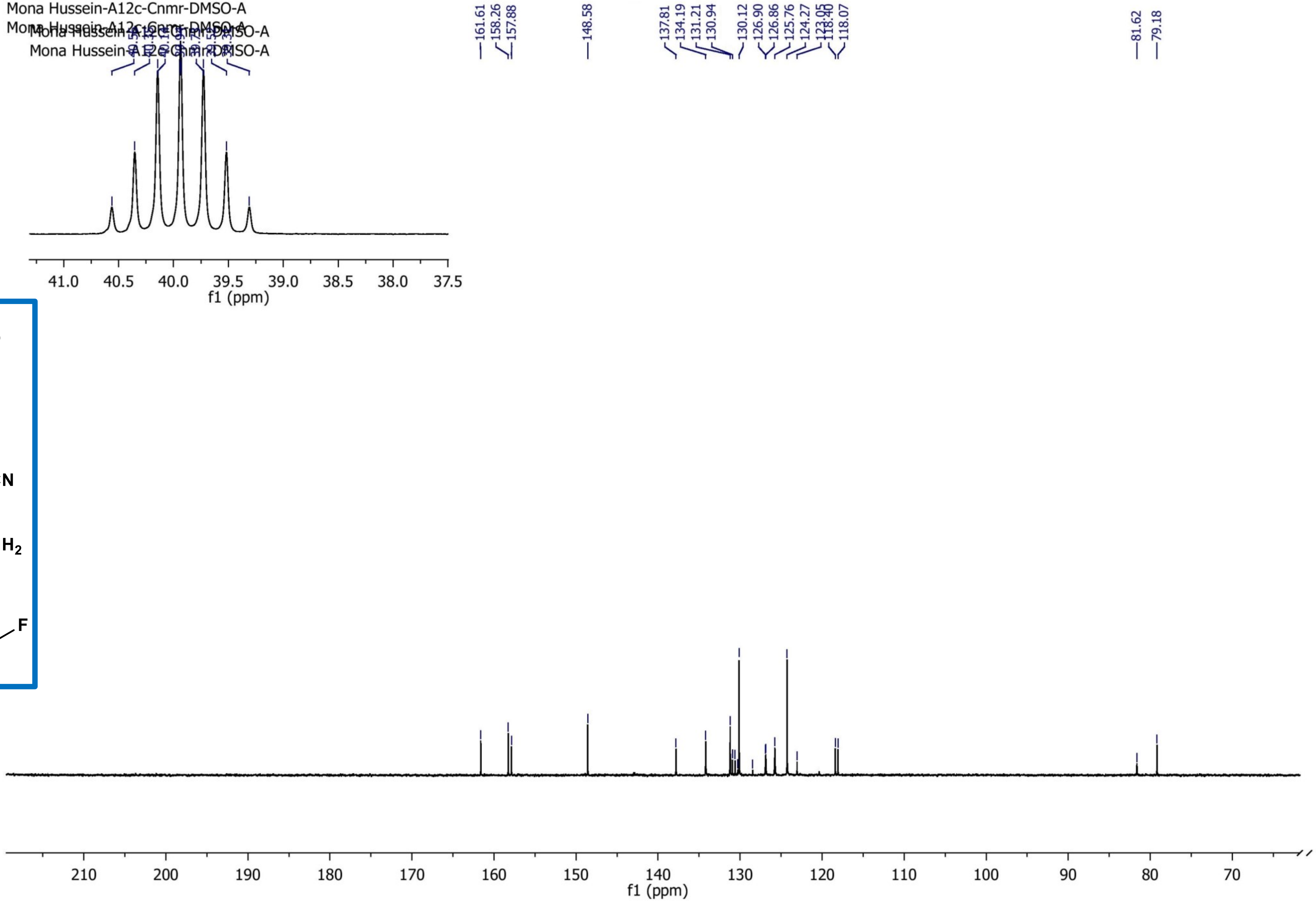
Mona Hussein-A12c-Cnmr-DMSO-A
161.61
158.26
157.88
148.58
137.81
134.19
131.21
130.94
130.12
126.90
126.86
125.76
124.27
123.05
118.40
118.07
81.62
79.18
41.0 40.5 40.0 39.5 39.0 38.5 38.0 37.5
f1 (ppm)
210 200 190 180 170 160 150 140 130 120 110 100 90 80 70
f1 (ppm)

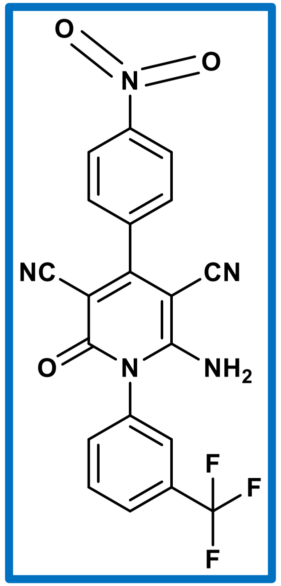
O
N
O
NC
CN
O
N
NH2
F
F
F

5c

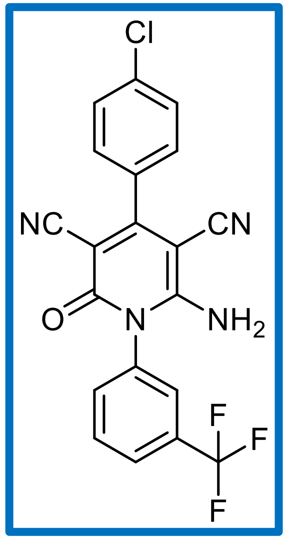
Cl
NC
CN
O
N
NH2
F
F
F

5d

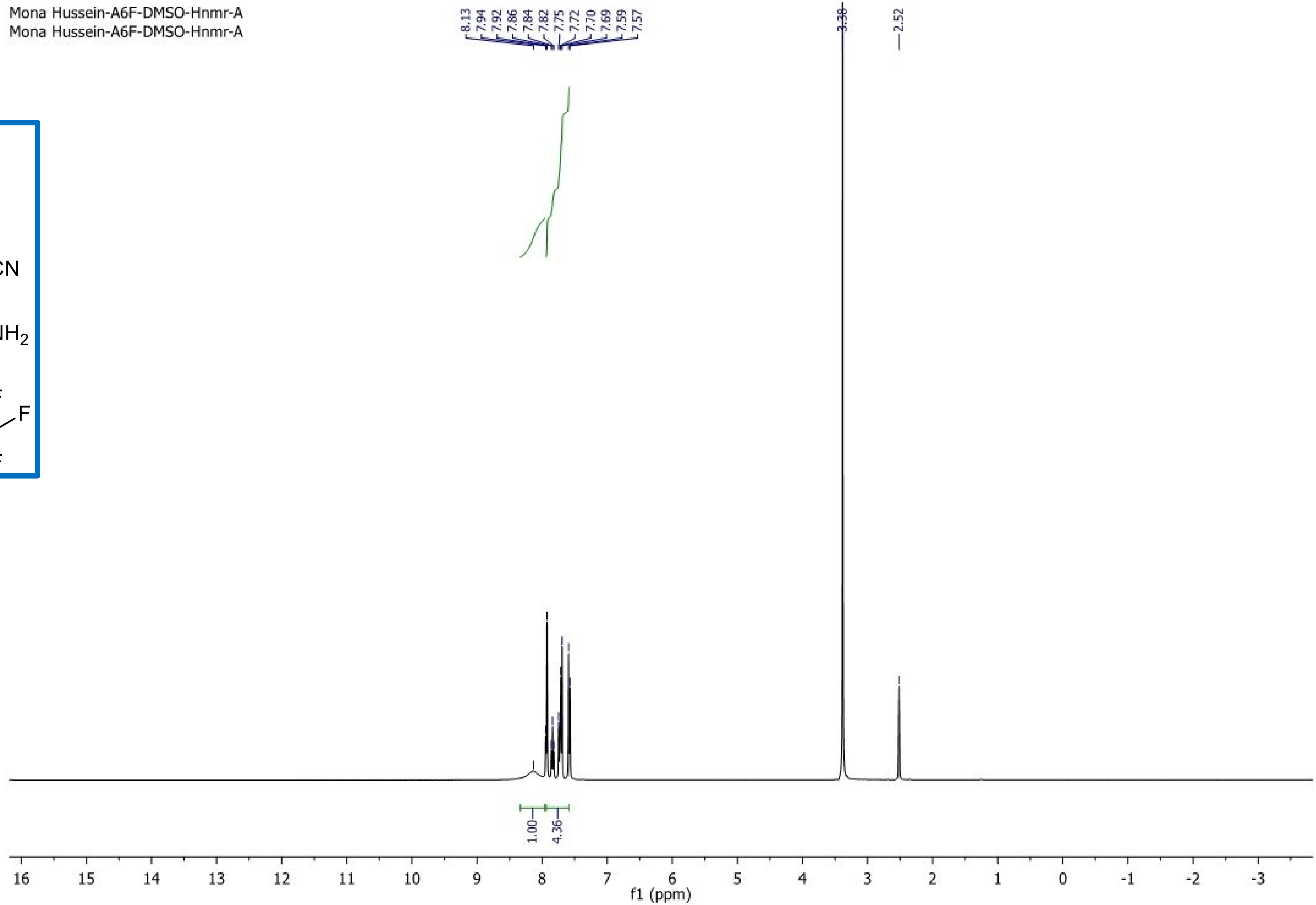
Mona Hussein-A6F-DMSO-Hnmr-A
Mona Hussein-A6F-DMSO-Hnmr-A
8.13
7.94
7.92
7.86
7.84
7.82
7.75
7.72
7.70
7.69
7.59
7.57
3.38
2.52
1.00
4.36
16 15 14 13 12 11 10 9 8 7 6 5 4 3 2 1 0 -1 -2 -3
f1 (ppm)

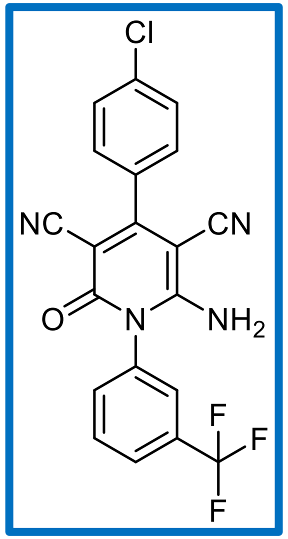
Cl
NC
CN
O
N
NH2
F
F
F

5d

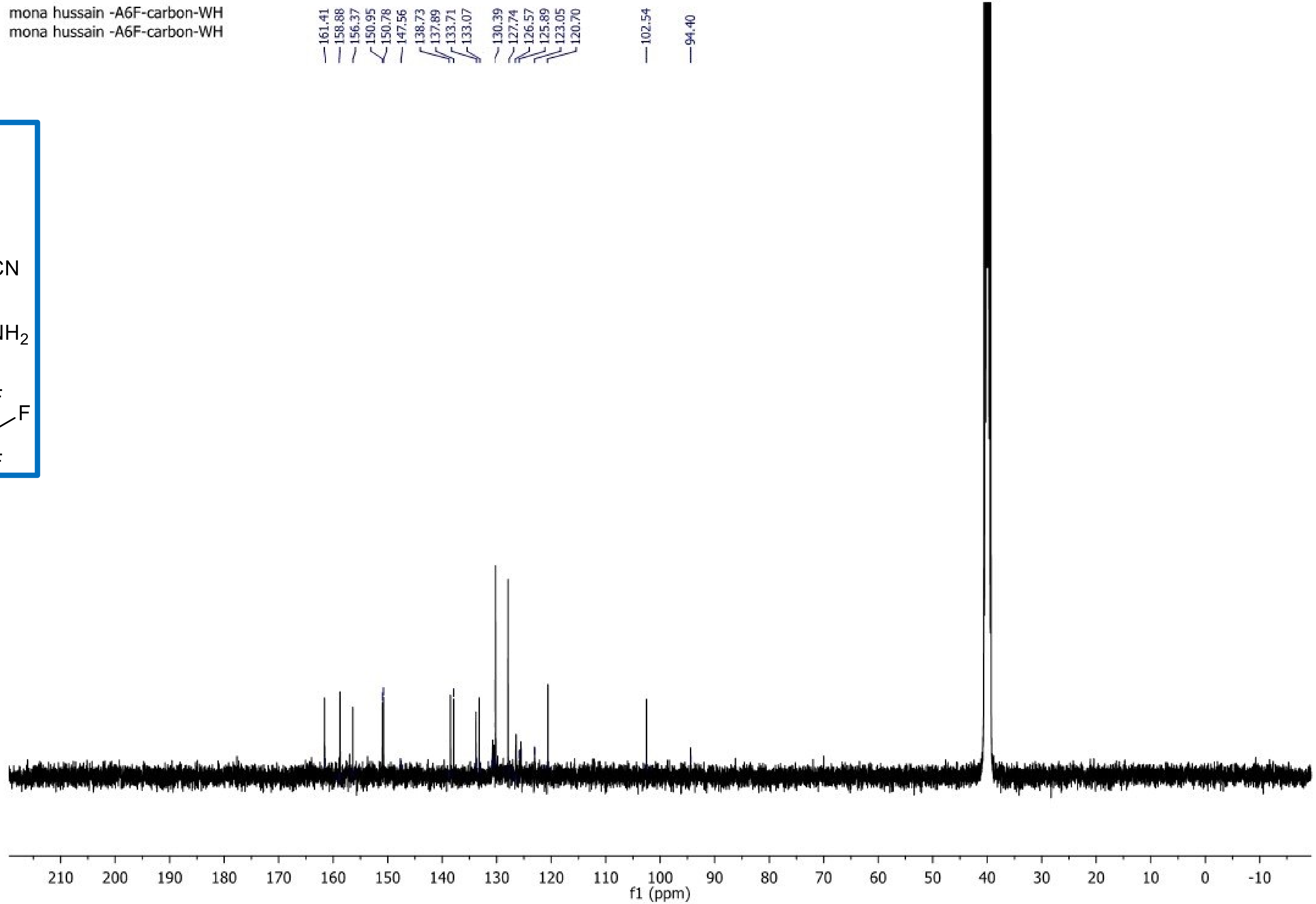
mona hussain -A6F-carbon-WH
mona hussain -A6F-carbon-WH
161.41
158.88
156.37
150.95
150.78
147.56
138.73
137.89
133.71
133.07
130.39
127.74
126.57
125.89
123.05
120.70
102.54
94.40
210
200
190
180
170
160
150
140
130
120
110
100
90
80
70
60
50
40
30
20
10
0
-10
f1 (ppm)

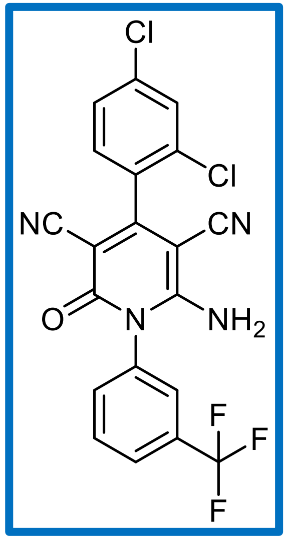
Cl
Cl
NC
CN
O
N
NH2
F
F
F

5e

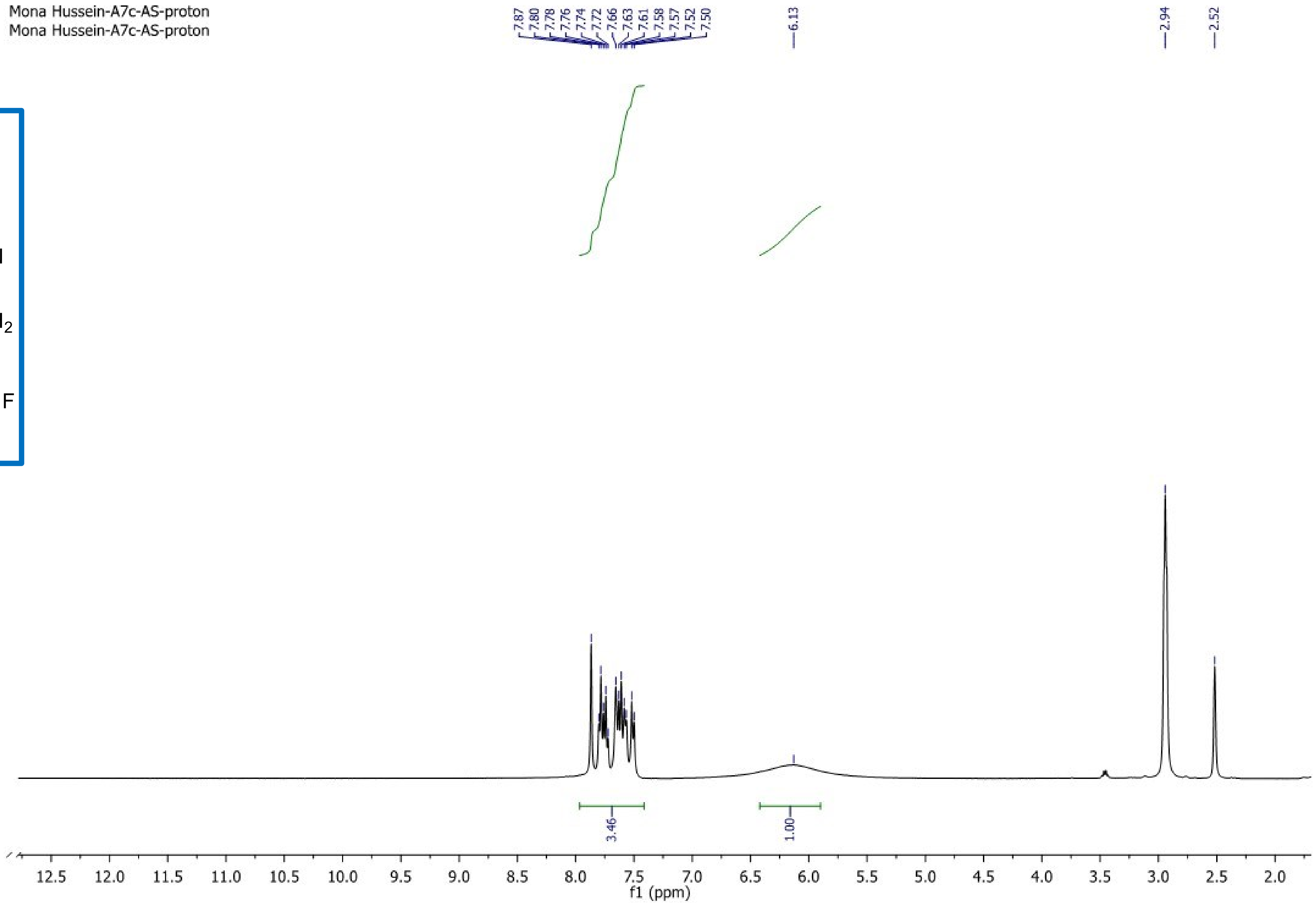
Mona Hussein-A7c-AS-proton
Mona Hussein-A7c-AS-proton
7.87
7.80
7.78
7.76
7.74
7.72
7.66
7.63
7.61
7.58
7.57
7.52
7.50
6.13
2.94
2.52
3.46
1.00
12.5
12.0
11.5
11.0
10.5
10.0
9.5
9.0
8.5
8.0
7.5
7.0
6.5
6.0
5.5
5.0
4.5
4.0
3.5
3.0
2.5
2.0
f1 (ppm)

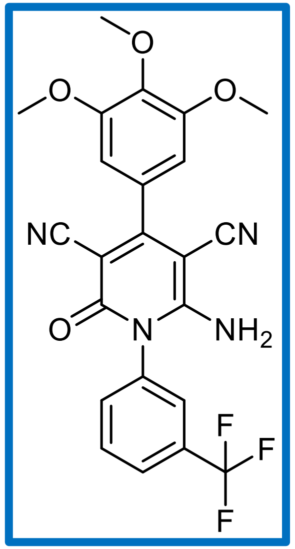

O
O
O
NC
CN
O
N
NH2
F
F
F

5f

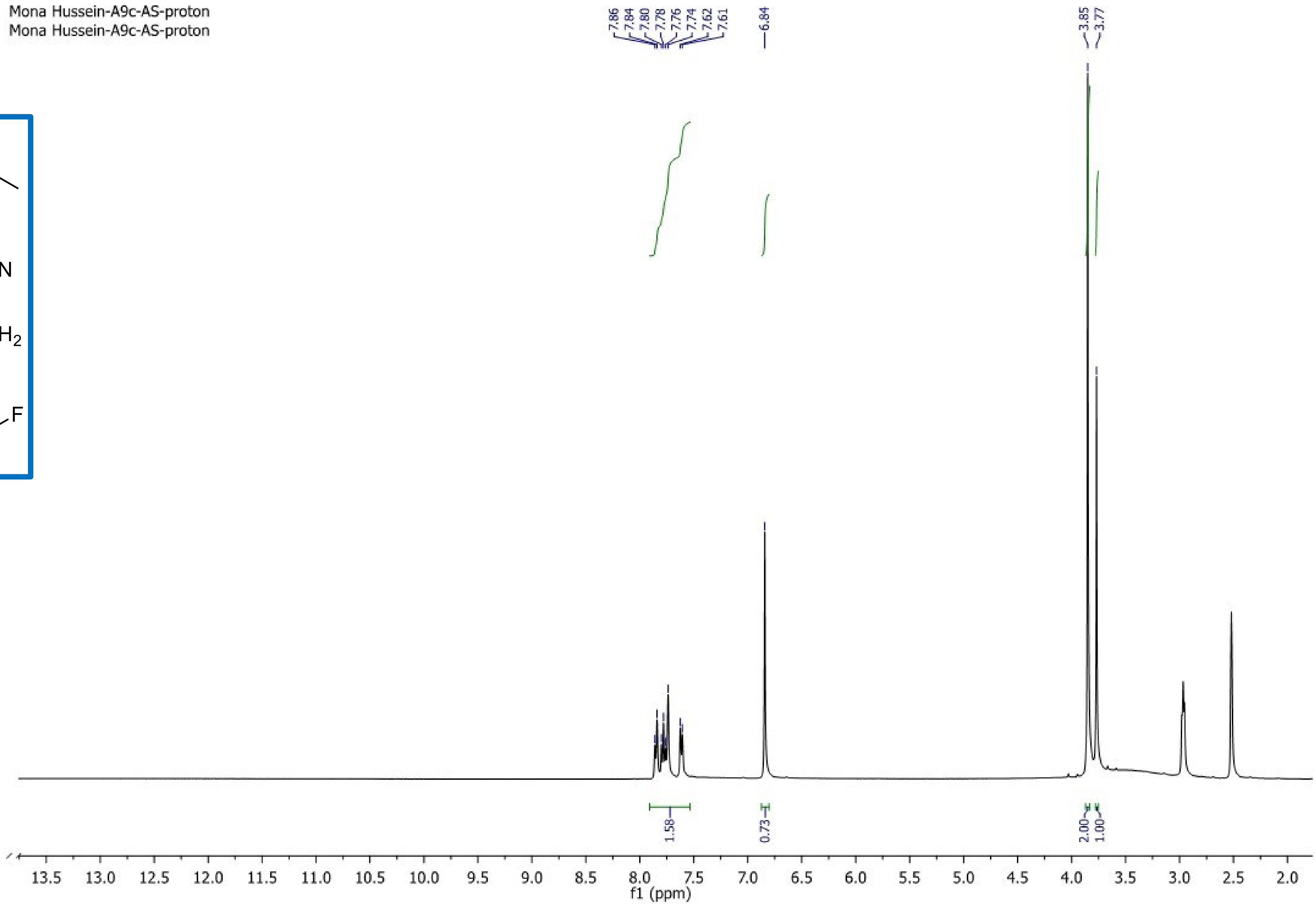

Mona Hussein-A9c-AS-proton
Mona Hussein-A9c-AS-proton
7.86
7.84
7.80
7.78
7.76
7.74
7.62
7.61
6.84
3.85
3.77
1.58
0.73
2.00
1.00
13.5 13.0 12.5 12.0 11.5 11.0 10.5 10.0 9.5 9.0 8.5 8.0 7.5 7.0 6.5 6.0 5.5 5.0 4.5 4.0 3.5 3.0 2.5 2.0
f1 (ppm)

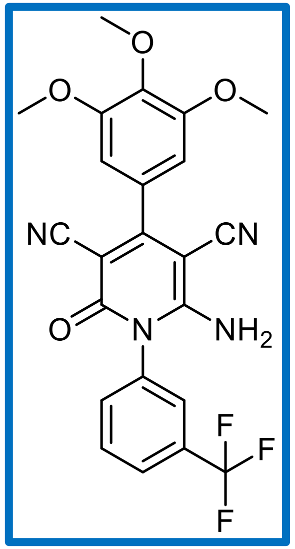
NC
CN
O
N
NH2
F
F
F
O
O
O

5f

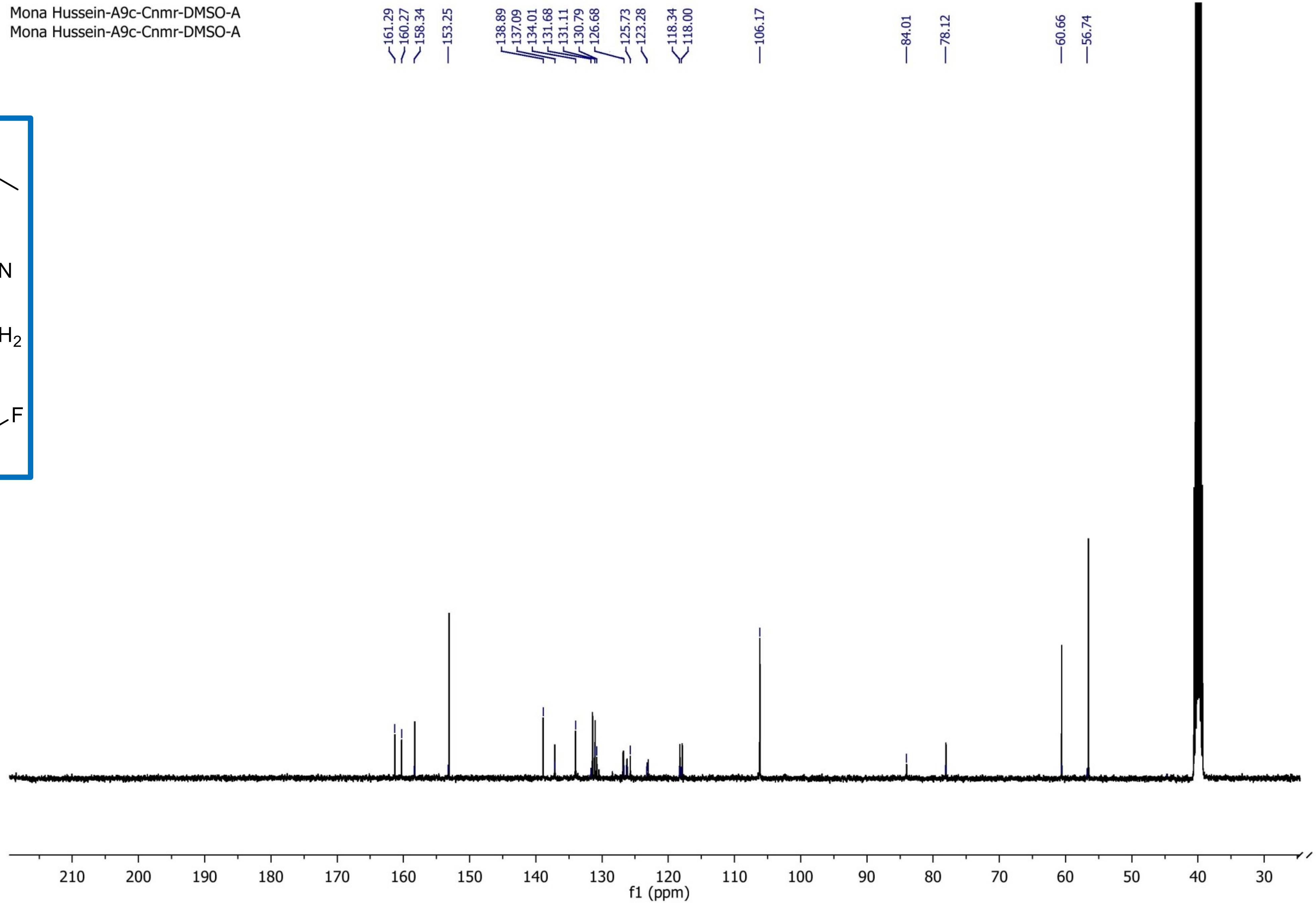
Mona Hussein-A9c-Cnmr-DMSO-A
Mona Hussein-A9c-Cnmr-DMSO-A
161.29
160.27
158.34
153.25
138.89
137.09
134.01
131.68
131.11
130.79
126.68
125.73
123.28
118.34
118.00
106.17
84.01
78.12
60.66
56.74
210
200
190
180
170
160
150
140
130
120
110
100
90
80
70
60
50
40
30
f1 (ppm)

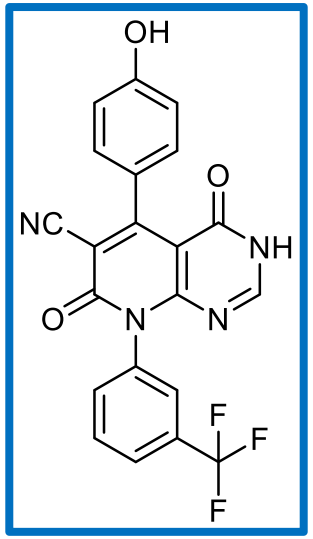

6a

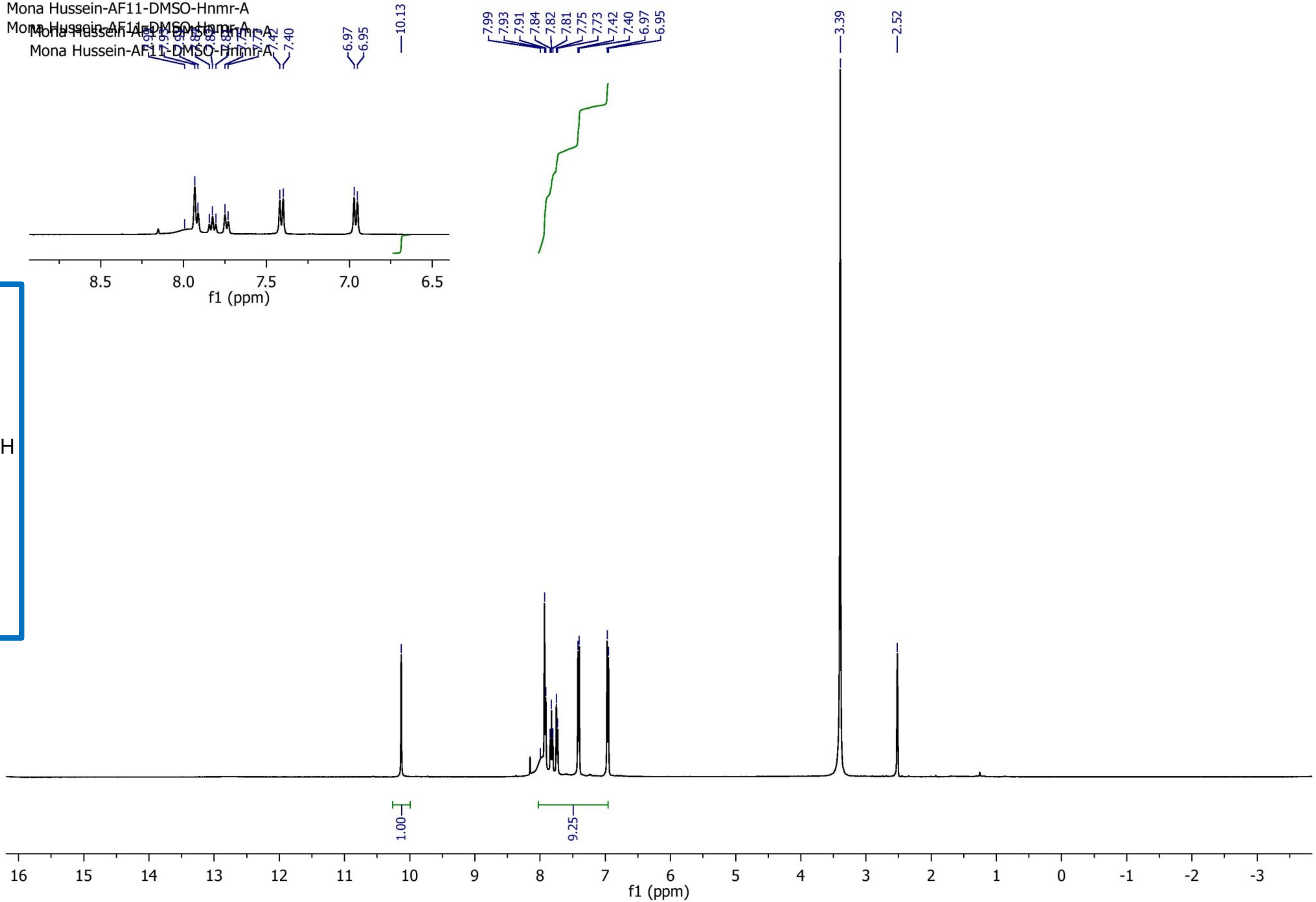
Mona Hussein-AF11-DMSO-Hnmr-A
10.13
7.99
7.93
7.91
7.84
7.82
7.81
7.75
7.73
7.42
7.40
6.97
6.95
3.39
2.52
1.00
9.25
f1 (ppm)

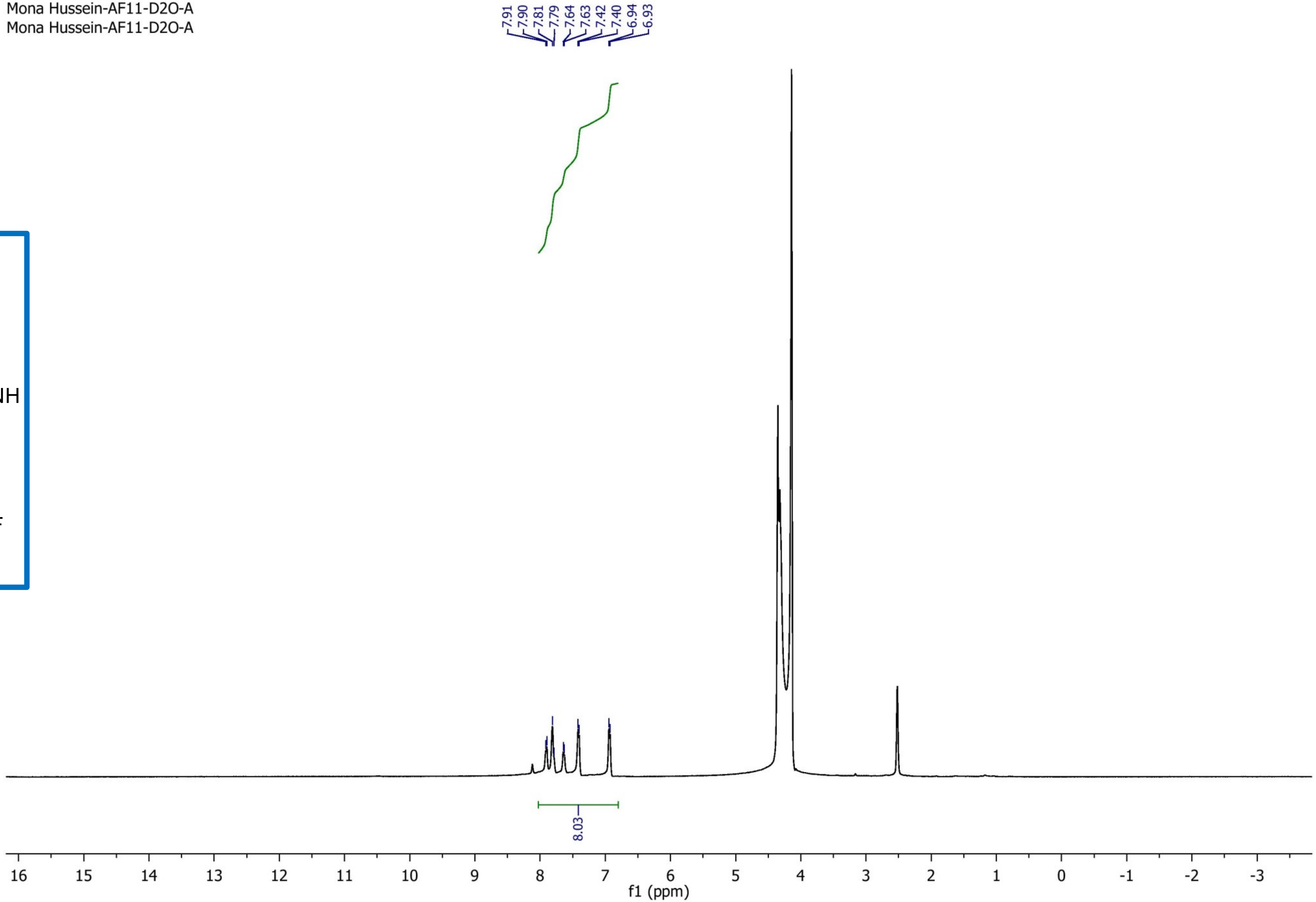
Mona Hussein-AF11-D2O-A
Mona Hussein-AF11-D2O-A
7.91
7.90
7.81
7.79
7.64
7.63
7.42
7.40
6.94
6.93
8.03
16 15 14 13 12 11 10 9 8 7 6 5 4 3 2 1 0 -1 -2 -3
f1 (ppm)

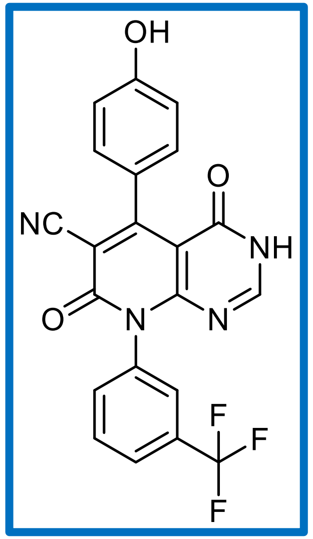
OH
O
NC
NH
O
N
N
F
F
F

6a

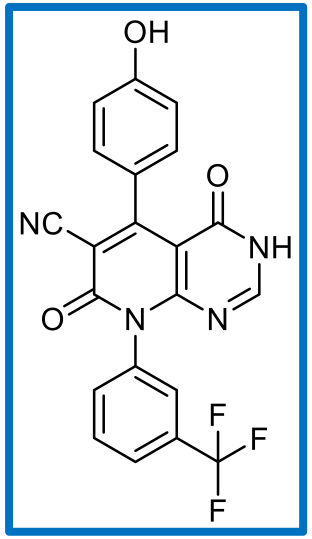

OH
O
NC
NH
O
N
N
F
F
F

6a

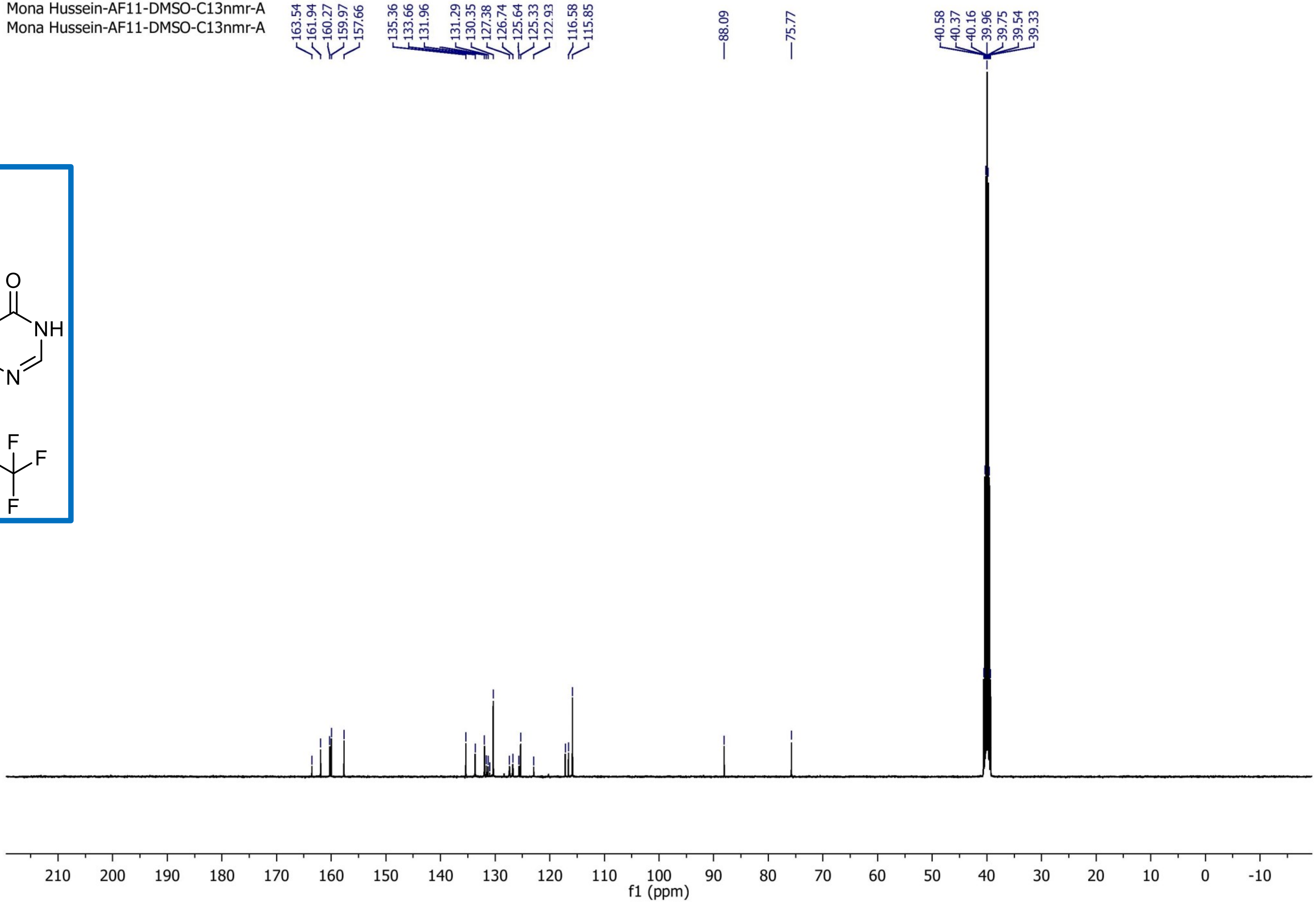

Mona Hussein-AF11-DMSO-C13nmr-A
Mona Hussein-AF11-DMSO-C13nmr-A
163.54
161.94
160.27
159.97
157.66
135.36
133.66
131.96
131.29
130.35
127.38
126.74
125.64
125.33
122.93
116.58
115.85
88.09
75.77
40.58
40.37
40.16
39.96
39.75
39.54
39.33
210
200
190
180
170
160
150
140
130
120
110
100
90
80
70
60
50
40
30
20
10
0
-10
f1 (ppm)

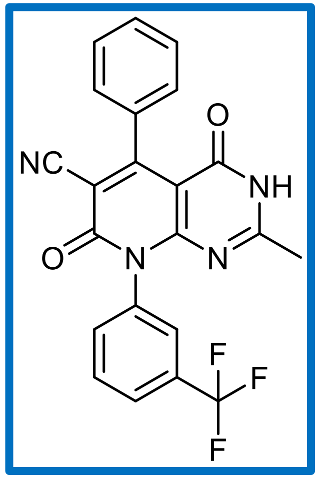
NC
O
NH
O
N
N
F
F
F

7a

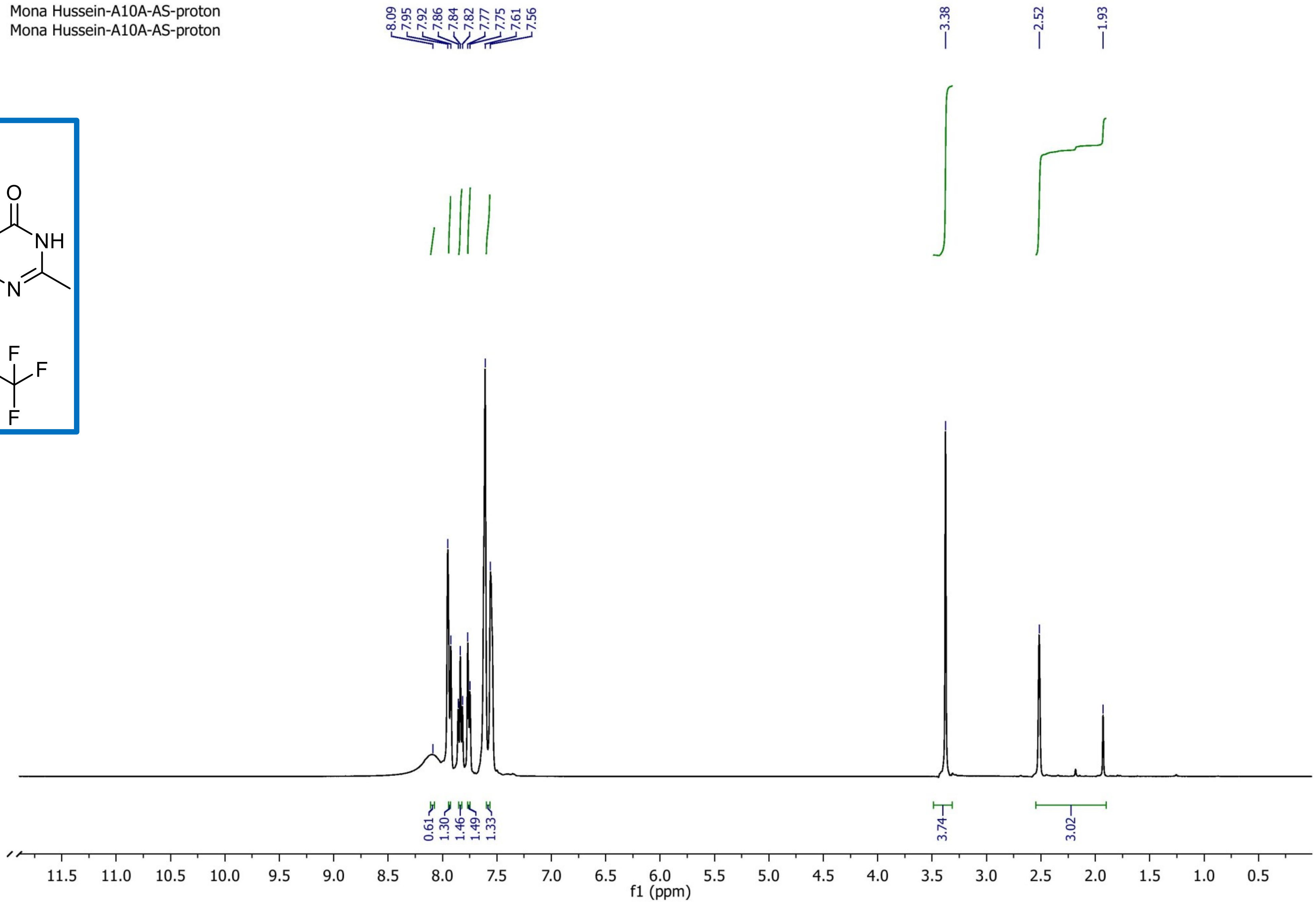
Mona Hussein-A10A-AS-proton
Mona Hussein-A10A-AS-proton
8.09
7.95
7.92
7.86
7.84
7.82
7.77
7.75
7.61
7.56
3.38
2.52
1.93
0.61
1.30
1.46
1.49
1.33
3.74
3.02
11.5 11.0 10.5 10.0 9.5 9.0 8.5 8.0 7.5 7.0 6.5 6.0 5.5 5.0 4.5 4.0 3.5 3.0 2.5 2.0 1.5 1.0 0.5
f1 (ppm)

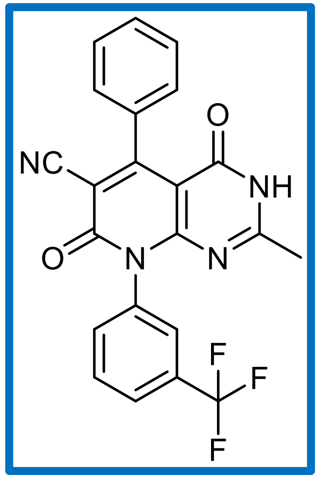
NC
O
NH
O
N
N
F
F
F

7a

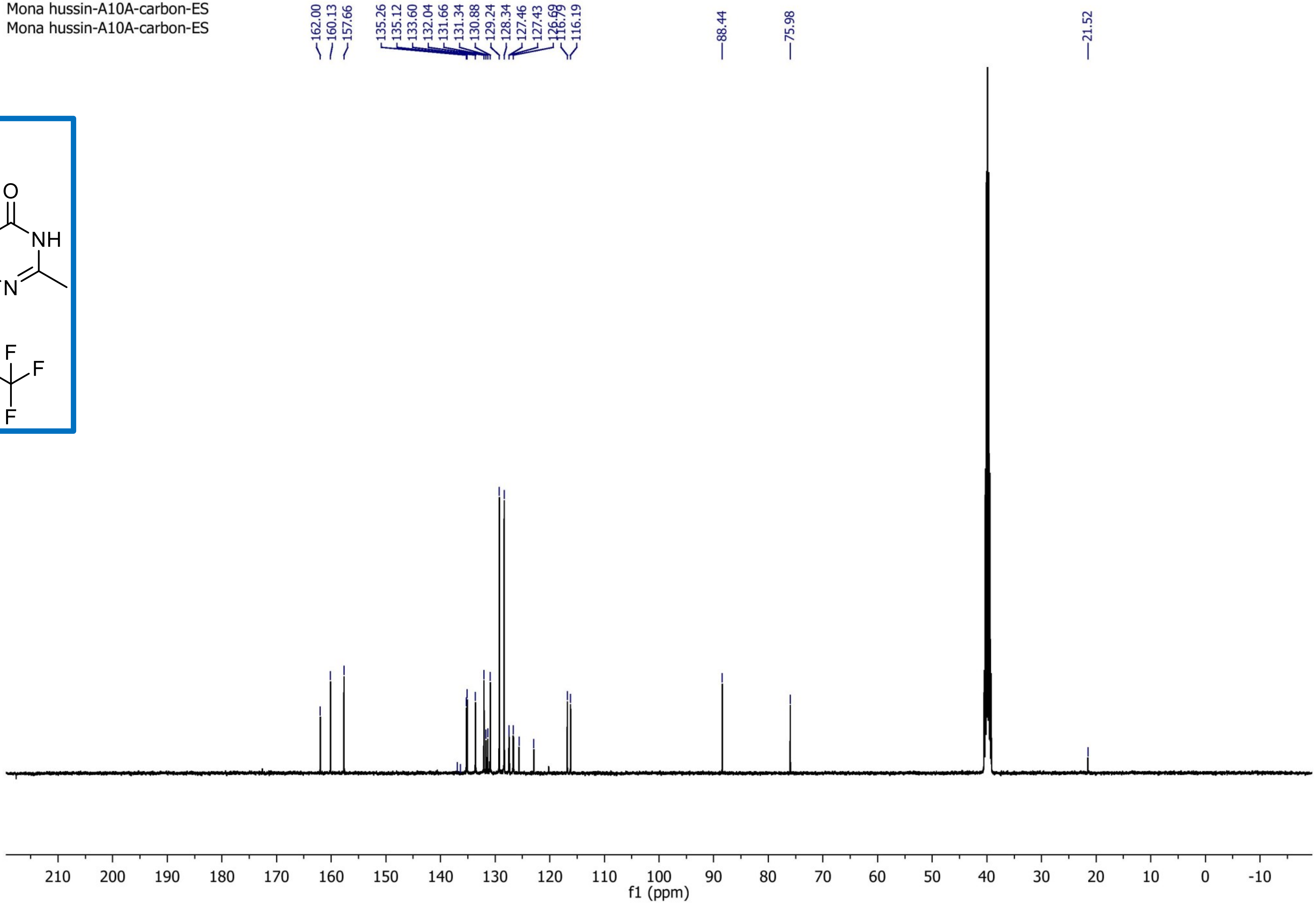
Mona hussin-A10A-carbon-ES
Mona hussin-A10A-carbon-ES
162.00
160.13
157.66
135.26
135.12
133.60
132.04
131.66
131.34
130.88
129.24
128.34
127.46
127.43
126.69
116.79
116.19
88.44
75.98
21.52
210
200
190
180
170
160
150
140
130
120
110
100
90
80
70
60
50
40
30
20
10
0
-10
f1 (ppm)

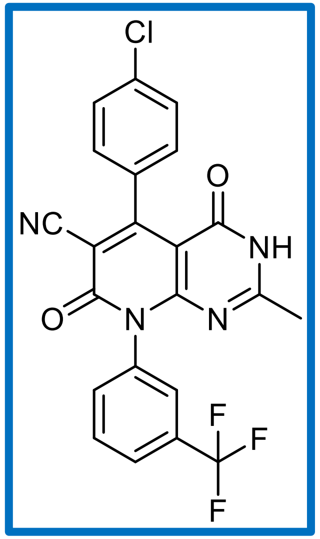
Cl
O
NC
NH
O
N
N
F
F
F

7b

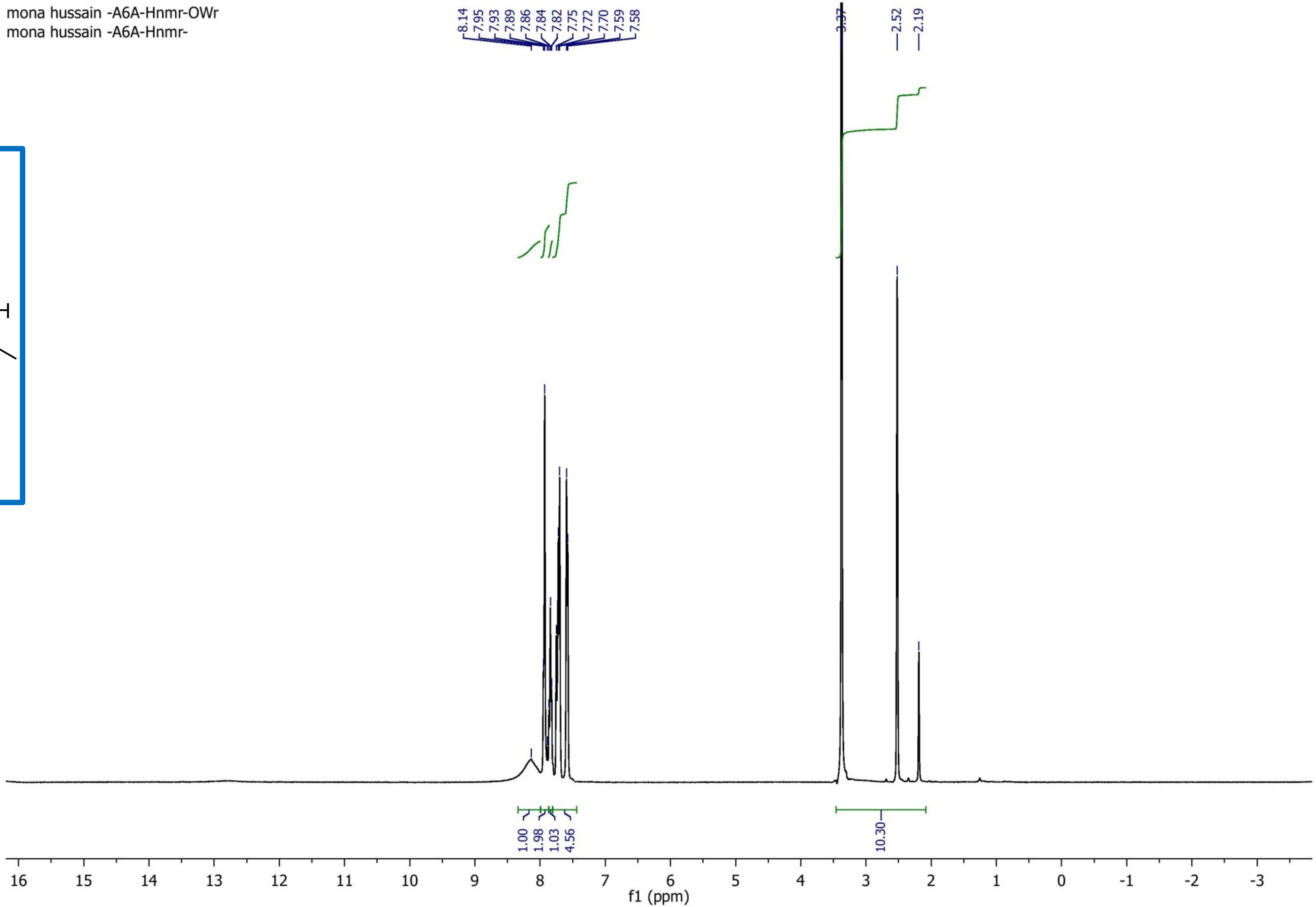
mona hussain -A6A-Hnmr-OWr
mona hussain -A6A-Hnmr-
8.14
7.95
7.93
7.89
7.86
7.84
7.82
7.75
7.72
7.70
7.59
7.58
3.37
2.52
2.19
1.00
1.98
1.03
4.56
10.30
16 15 14 13 12 11 10 9 8 7 6 5 4 3 2 1 0 -1 -2 -3
f1 (ppm)

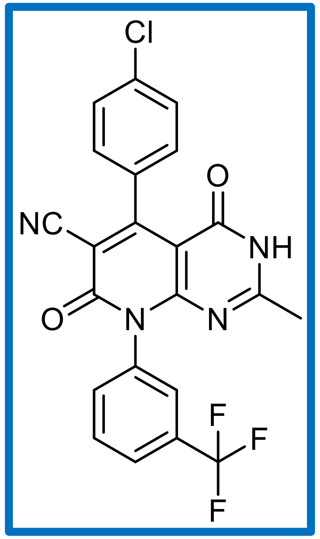
Cl
O
NC
NH
O
N
N
F
F
F

7b

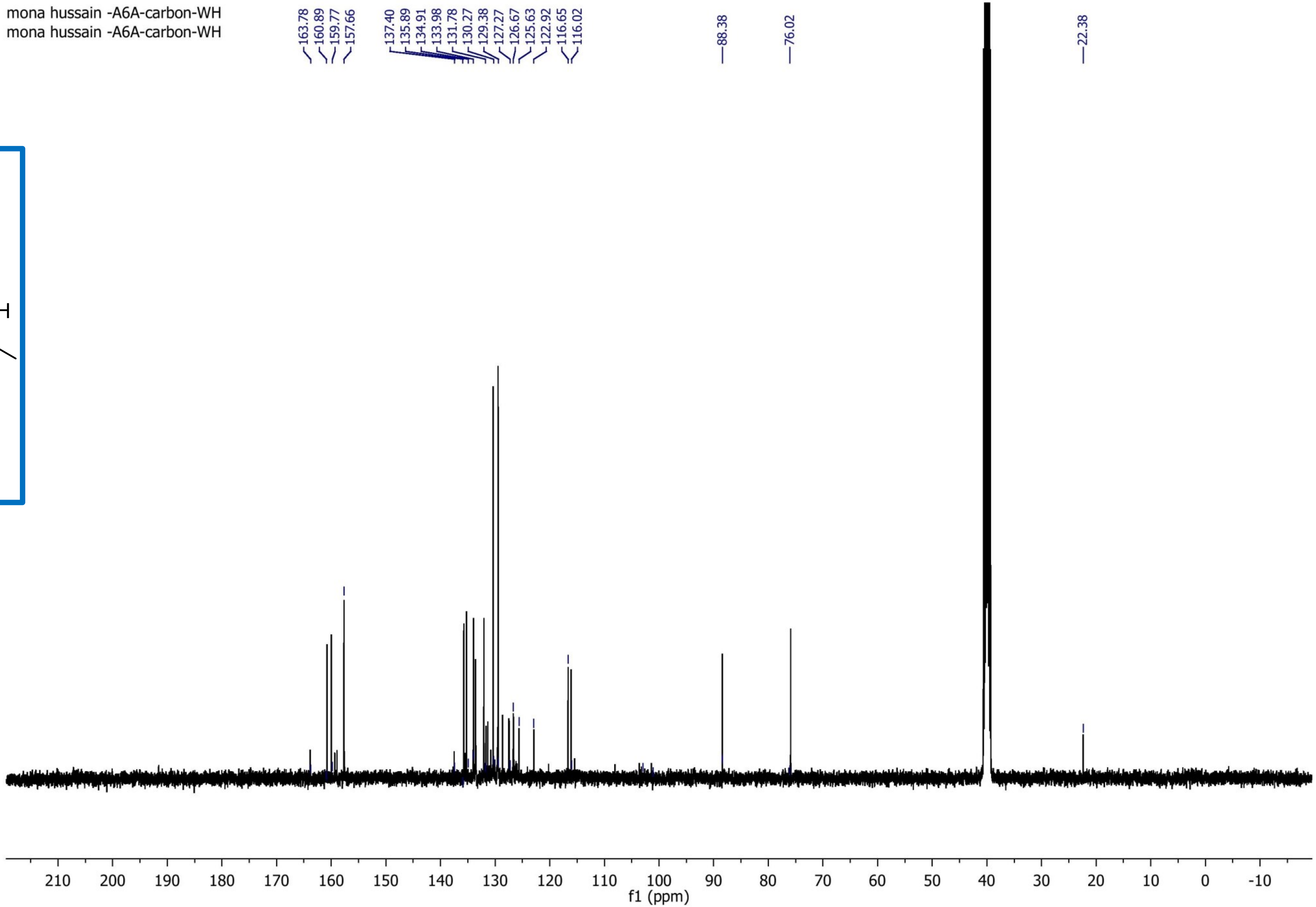
mona hussain -A6A-carbon-WH
mona hussain -A6A-carbon-WH
163.78
160.89
159.77
157.66
137.40
135.89
134.91
133.98
131.78
130.27
129.38
127.27
126.67
125.63
122.92
116.65
116.02
88.38
76.02
22.38
210
200
190
180
170
160
150
140
130
120
110
100
90
80
70
60
50
40
30
20
10
0
-10
f1 (ppm)

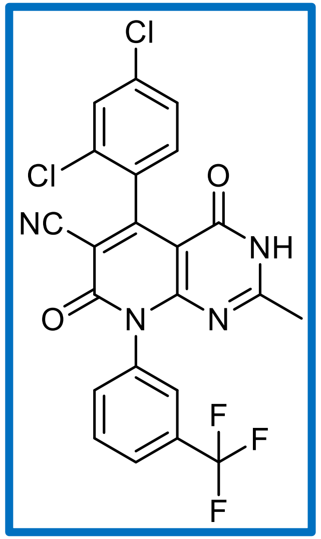

7c

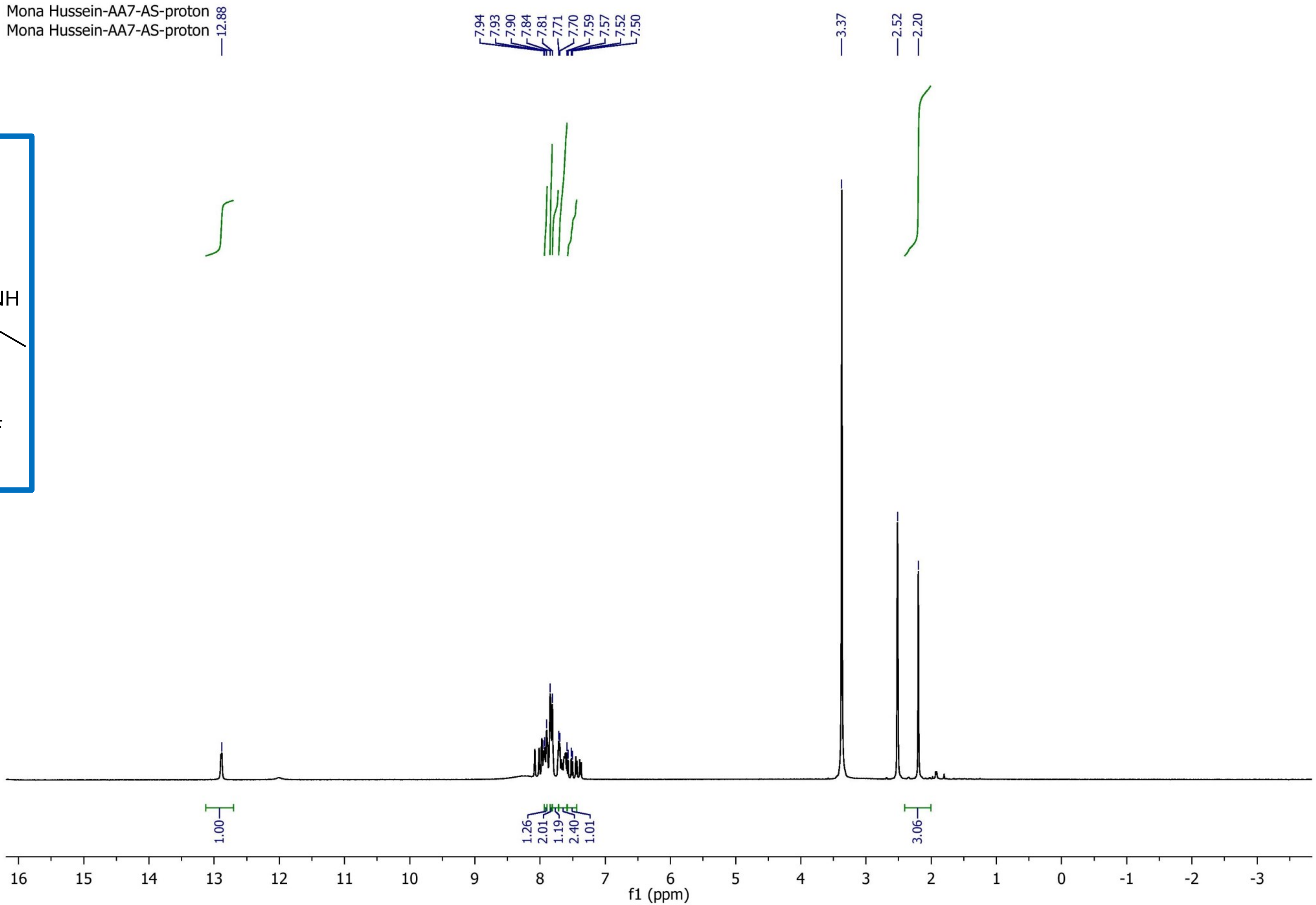
Mona Hussein-AA7-AS-proton
Mona Hussein-AA7-AS-proton
12.88
7.94
7.93
7.90
7.84
7.81
7.71
7.70
7.59
7.57
7.52
7.50
3.37
2.52
2.20
1.00
1.26
2.01
1.19
2.40
1.01
3.06
16
15
14
13
12
11
10
9
8
7
6
5
4
3
2
1
0
-1
-2
-3
f1 (ppm)

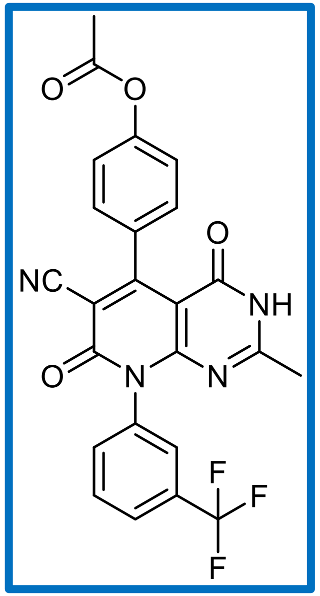
O
O
O
NC
NH
O
N
N
F
F
F

7d

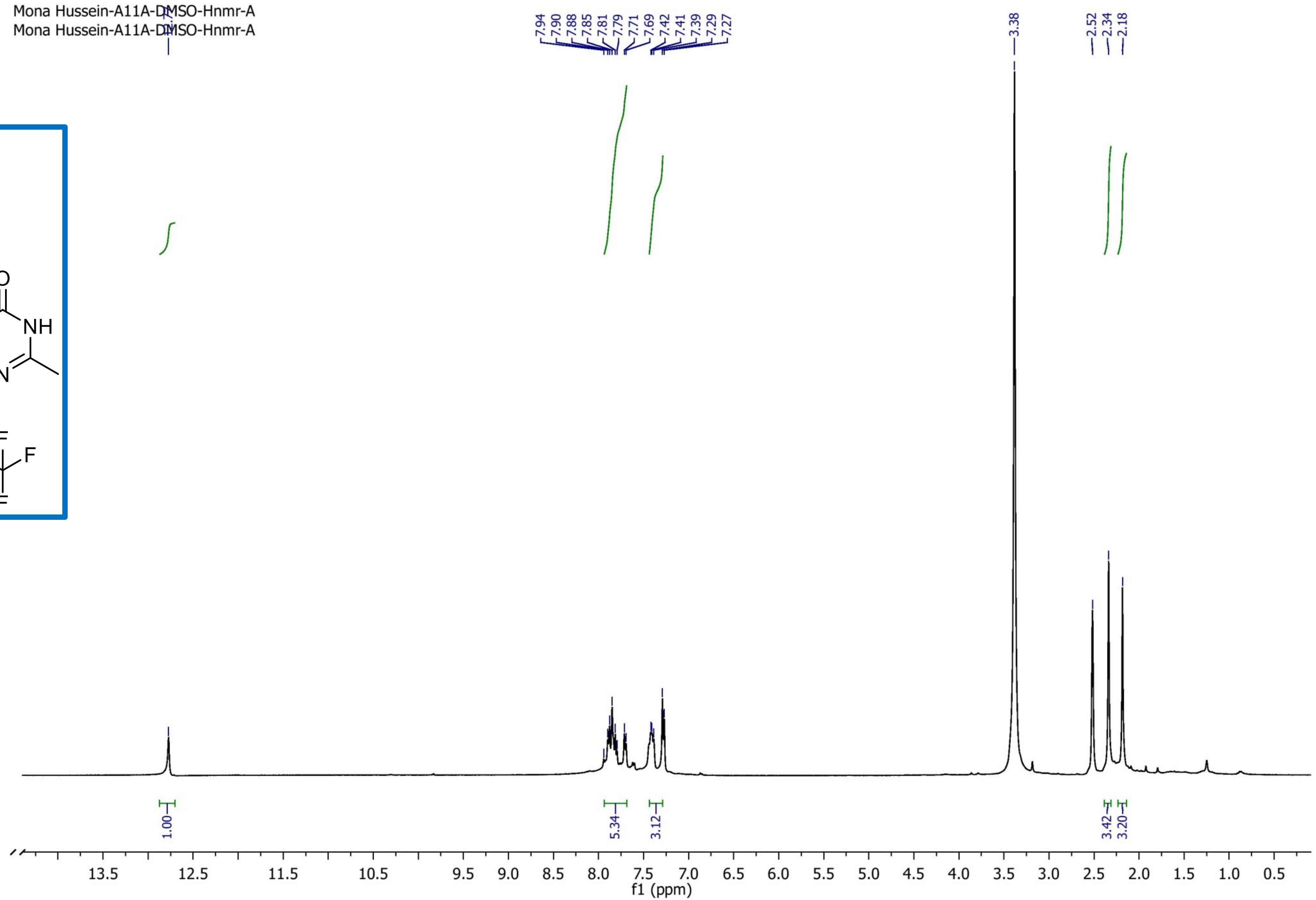
Mona Hussein-A11A-DMSO-Hnmr-A
Mona Hussein-A11A-DMSO-Hnmr-A
7.94
7.90
7.88
7.85
7.81
7.79
7.71
7.69
7.42
7.41
7.39
7.29
7.27
3.38
2.52
2.34
2.18
1.00
5.34
3.12
3.42
3.20
13.5
12.5
11.5
10.5
9.5
9.0
8.5
8.0
7.5
7.0
6.5
6.0
5.5
5.0
4.5
4.0
3.5
3.0
2.5
2.0
1.5
1.0
0.5
f1 (ppm)

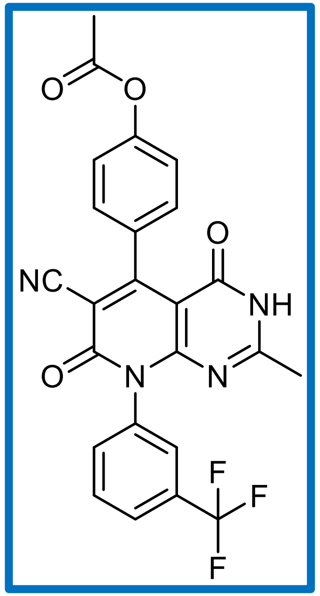
O
O
O
NC
NH
N
N
F
F
F

7d

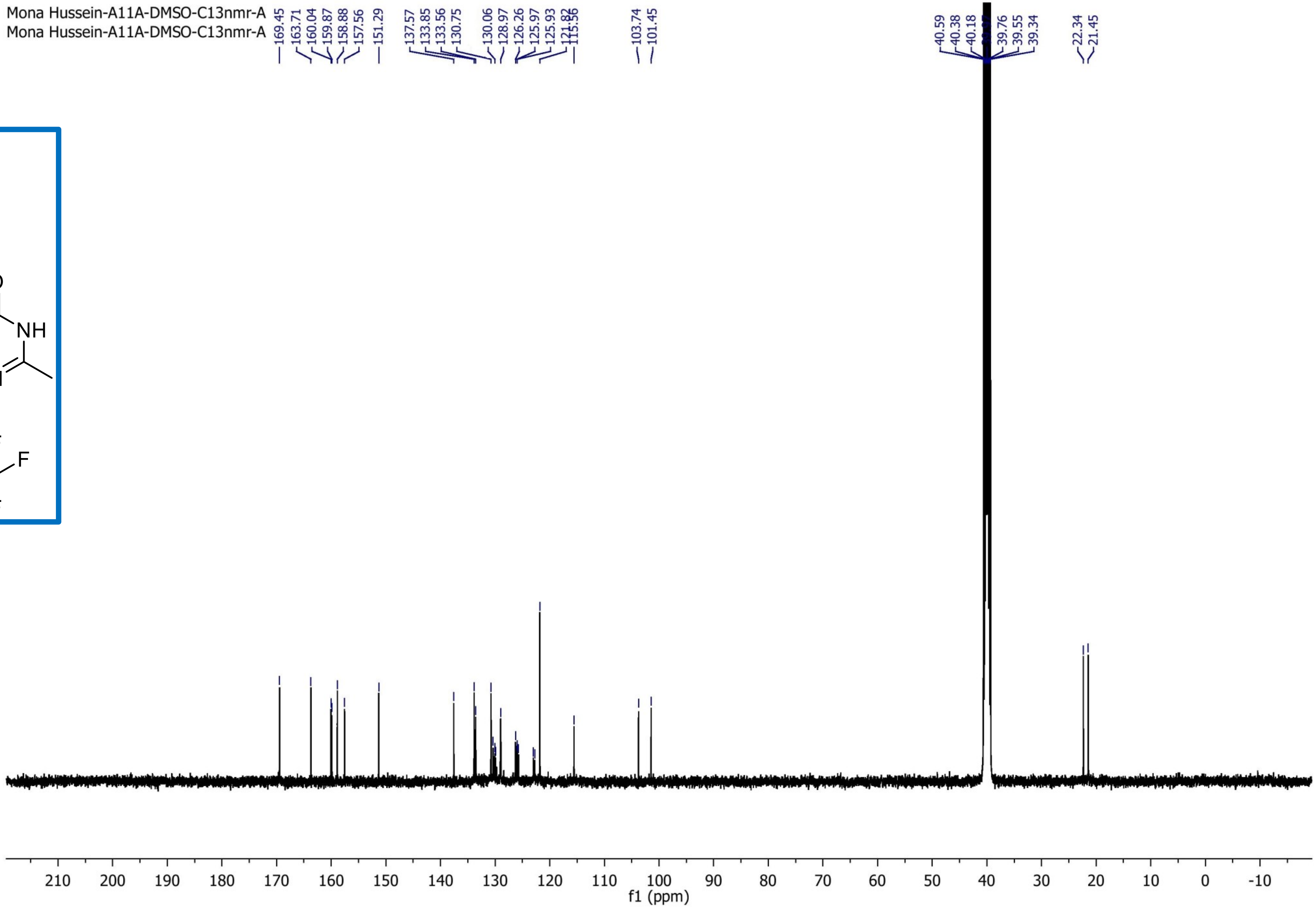
Mona Hussein-A11A-DMSO-C13nmr-A
Mona Hussein-A11A-DMSO-C13nmr-A
169.45
163.71
160.04
159.87
158.88
157.56
151.29
137.57
133.85
133.56
130.75
130.06
128.97
126.26
125.97
125.93
121.82
115.56
103.74
101.45
40.59
40.38
40.18
39.76
39.55
39.34
22.34
21.45
210
200
190
180
170
160
150
140
130
120
110
100
90
80
70
60
50
40
30
20
10
0
-10
f1 (ppm)
